# Correlations Between Vibrational Red Shifts, Electronic Structure, and Water Dissociation on Alloy Clusters: An Exploratory Computational Study

**DOI:** 10.3390/molecules31183165

**Published:** 2026-09-08

**Authors:** Yanbiao Wang, Xinglong Pan, Tingting Liu

**Affiliations:** 1 Department of Fundamental Courses, Wuxi University of Technology, Wuxi 214121, China; 2 Department of Physics, Southeast University, Nanjing 211189, China; 3 School of Mechanical Technology, Wuxi University of Technology, Wuxi 214121, China

**Keywords:** water dissociation, first-principles molecular dynamics, catalysis, vibrational infrared spectroscopy, frontier orbitals, resonant absorption

## Abstract

Understanding the activation mechanism of water molecules is of fundamental importance for catalytic water dissociation via water splitting. In this study, we employed first-principles molecular dynamics simulations to investigate the catalytic dissociation of H_2_O on 88 alloy clusters. Our results reveal that a larger red shift in the center of the coupled ν_1_ and ν_3_ stretching modes of adsorbed H_2_O correlates with a lower dissociation temperature. We observe an empirical correlation suggesting that dissociation is facilitated when the adsorption energy is comparable in magnitude to the HOMO–LUMO gap, which we propose as a hypothesis warranting further investigation with excited-state methods. Furthermore, comparison of the frontier molecular orbitals between precursor and intermediate states demonstrates that the number of frontier orbitals exhibiting increased overlap with the dissociating H atom is inversely correlated with the dissociation temperature. These findings provide atomic-scale insights into the activation mechanism of water dissociation on isolated gas-phase clusters and establish spectroscopic descriptors for evaluating the intrinsic reactivity of such model systems. We caution that extension of these findings to practical catalytic water splitting would require consideration of additional factors such as catalyst supports, solvents, and realistic reaction conditions.

## 1. Introduction

Hydrogen is widely regarded as a promising energy carrier for addressing global warming, environmental pollution, and the fossil energy crisis, provided it is produced through sustainable pathways [1,2,3]. The overall reaction for hydrogen production via water splitting is thermodynamically uphill, with a positive Gibbs free energy change of ΔG° = 237.13 kJ/mol, and typically cannot proceed spontaneously due to substantial kinetic barriers. Catalysts with tunable electronic and optical properties at specific dimensions, shapes, and compositions can effectively reduce these energy barriers, thereby facilitating the reaction [4]. Since Fujishima and Honda first demonstrated photoelectrochemical water splitting using an n-type TiO_2_ photoanode and a Pt cathode under UV irradiation in 1972 [5], extensive research has been devoted to various materials for photocatalytic water splitting [6,7,8,9,10,11,12,13,14,15,16,17,18,19,20,21].

Water dissociation can be achieved through either electronic excitation [22,23] or vibrational excitation [24,25]. The water molecule exhibits three distinct vibrational normal modes: bending (*ν*_2_), symmetric stretching (*ν*_1_), and antisymmetric stretching (*ν*_3_). The two stretching modes are strongly coupled via Darling–Dennison resonance due to their close vibrational frequencies and anharmonicity, whereas the coupling between the bending mode and stretching motions is relatively weak [26,27]. According to Polanyi’s rules, a “late” barrier is more readily surmounted by vibrational excitation, while an “early” barrier is more effectively overcome by translational energy, as the promoting coordinate coincides with the reaction coordinate near the transition state [28]. The dissociative chemisorption of H_2_O features a late barrier, where bond cleavage occurs after molecular adsorption on the surface. Consequently, vibrational excitation of H_2_O can significantly influence reactivity. Vibrationally excited molecular hydrogen has been observed in dense photodominated regions [29]. Experimentally, it has been demonstrated that vibrational excitation enhances the reactivity of unimolecular and bimolecular reactions more effectively than translational or rotational excitation in the presence of a laser field [30,31].

Theoretical studies of water dissociative chemisorption have predicted strong mode specificity, bond selectivity, and steric effects on Cu(111) and Ni(111) surfaces [32,33,34,35]. In a pioneering study, Zhang et al. employed full-dimensional quantum dynamics to investigate H_2_O dissociative chemisorption on rigid Cu(111), revealing that vibrational excitations play a more pivotal role than increasing translational energy in promoting the reaction, with asymmetric stretching excitation exhibiting the most pronounced enhancement [36]. Tiwari and coworkers investigated the influence of alloying on mode-selectivity in H_2_O dissociation on Ni(100), Ni(110), Ni(111), and Cu/Ni bimetallic surfaces using a fully quantum approach based on the reaction path Hamiltonian. Their results demonstrated that vibrational excitation consistently leads to significant enhancement in reactivity across all systems [37,38], in agreement with quantum state-resolved molecular beam experiments on heavy water dissociation on Ni(111) [39]. The vibrational enhancement of reactivity can be understood within the sudden vector projection (SVP) model, where the mode-specific enhancement reflects its coupling with the reaction coordinate at the transition state [40,41]. Experimentally, Kim et al. successfully manipulated the state-selective dissociation of single water molecules using inelastic tunneling electrons with a low-temperature scanning tunneling microscope, achieving dissociation into hydroxyl via vibrational excitation of stretching modes and into atomic oxygen via electronic excitation [42]. Additionally, vibrationally excited hydrolysis dissociation experiments provide an effective means of determining dissociation thresholds. For instance, Maksyutenko et al. accurately determined the first dissociation threshold of water, *D*_0_ = 41,145.94 ± 0.15 cm^−1^, from the onset of the dissociative continuum in triple-resonance spectra [43].

Atomic-scale insights into the interactions of water molecules with catalysts are essential for understanding the elementary processes of hydrogen generation. Density functional theory (DFT) calculations have been widely employed to elucidate the molecular-level mechanisms of water interaction with metal surfaces [33]. Despite substantial experimental and theoretical efforts, only a few studies have addressed the role of chemical shifts in the infrared spectra of adsorbed water molecules as descriptors of reactivity. Since vibrational spectroscopy provides information on molecular structure and bonding, we previously investigated the correlation between reactivity and chemical shifts in the infrared vibrational spectra of H_2_O on Cu(10 − *n*)Pt*_n_* and Cu(9 − *n*)Pd*_n_* alloy catalysts [44,45]. In those studies, we introduced the concept of the center of the ν_1_ and ν_3_ modes—defined as (*ν*_1_ × *I*_1_ + *ν*_3_ × *I*_3_)/(*I*_1_ + *I*_3_), where *I*_1_ and *I*_3_ are the intensities of the respective modes—to characterize water reactivity, owing to the strong coupling of these two stretching modes with the reaction coordinate. Our results indicated that the dissociation barrier of H_2_O is closely correlated with the red shift in the center of the ν_1_ and ν_3_ modes of adsorbed H_2_O: a larger red shift corresponds to a lower dissociation barrier. We therefore hypothesize that this red shift can serve as a descriptor for the activation properties of catalysts. In the present study, we systematically examine the chemical shifts in the infrared vibrational spectra of H_2_O adsorbed on 88 alloy clusters to test this hypothesis and develop a predictive understanding of catalytic activity.

It is important to emphasize that the present study focuses on the elementary step of water dissociation on isolated gas-phase clusters. While this provides valuable insights into intrinsic catalytic activity, practical water-splitting systems involve additional complexities—including catalyst supports, solvation effects, electrolyte environments, applied electrode potentials, counterions, and surface coverage—that are not captured in our model. The correlations and mechanistic interpretations presented here should therefore be regarded as a foundation for understanding intrinsic reactivity rather than as direct predictions for real catalytic systems.

## 2. Computational Details

All calculations were performed using the BIOVIA DMol^3^ program (2019) with the generalized gradient approximation (GGA) and the Perdew–Burke–Ernzerhof (PBE) functional [46]. DFT-based relativistic semicore pseudopotentials (DSPPs) [47] with the double numerical plus polarization (DNP) basis set and an orbital cutoff of 4.5 Å were employed. The DNP basis set and DSPP pseudopotential have been widely validated for transition-metal-containing systems and offer a good balance between computational cost and accuracy. Electronic smearing of 0.005 Ha was applied to facilitate SCF convergence, and the ground state was determined by comparing total energies of different spin multiplicities. The orbital isosurface value was set to 0.03 e/Å^3^ for all frontier orbital visualizations. Dispersion corrections were tested using the Grimme D3 method for selected systems and were found to have negligible effects on the reported correlations; therefore, dispersion corrections were not included in the production calculations. Infrared spectra were subsequently calculated.

**Figure 1:**
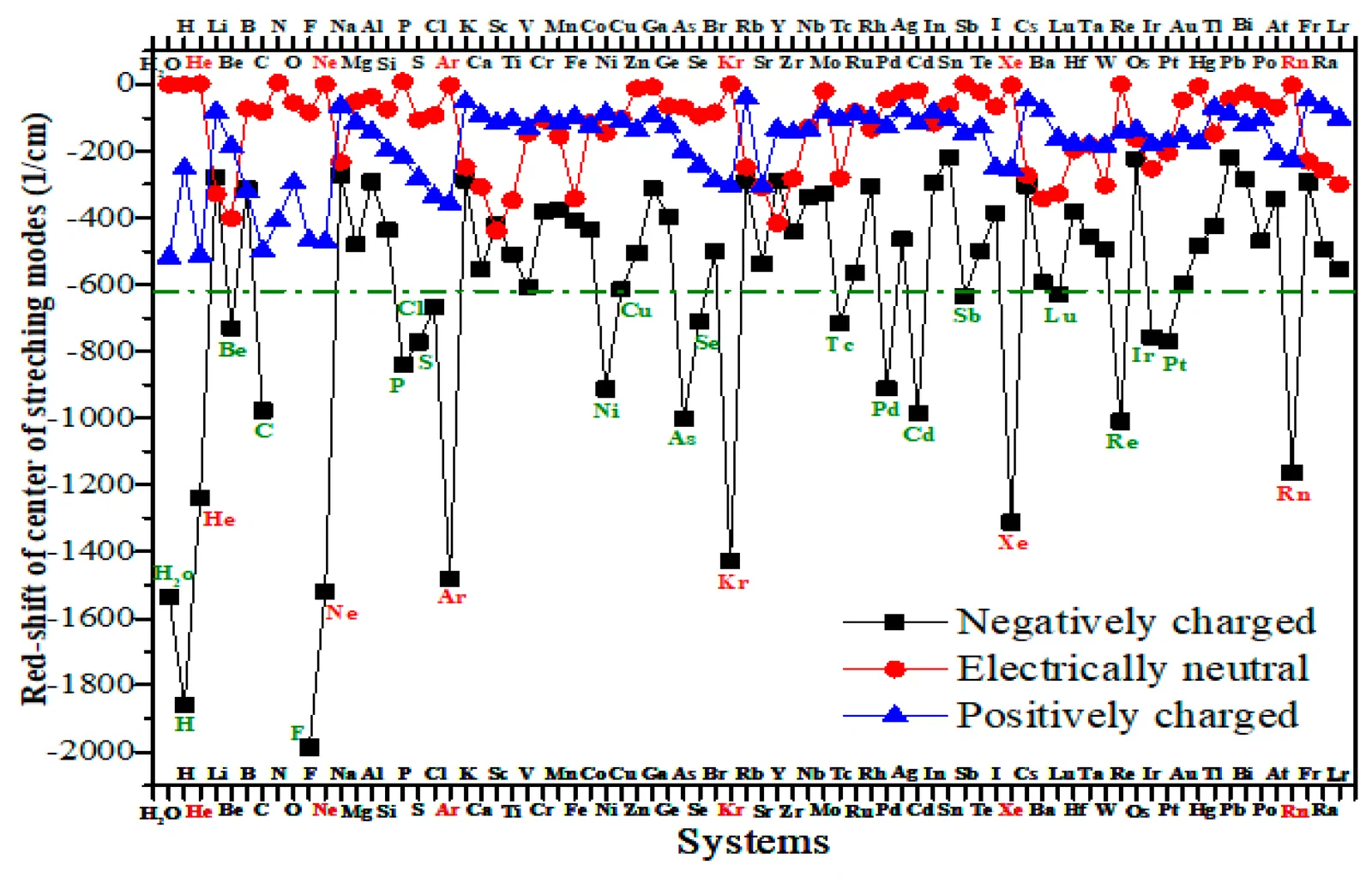
Red shifts of the centers of *ν*_1_ and *ν*_3_ modes of H_2_O adsorbed on the first 75 single atoms of the periodic table relative to isolated electrically neutral H_2_O. Black squares, red circles, and blue triangles represent negative, neutral, and positive charge states, respectively.

All calculations were performed with a total charge of −1. This choice was motivated by the observation that negatively charged systems exhibit the largest vibrational red shifts (Figure 1). The adsorption energy (*E*_a_) of H_2_O on each cluster was calculated using the following definition: *E*_a_ = *E*(H_2_O/cluster) − *E*(cluster) − *E*(H_2_O). Here, *E*(H_2_O/cluster) is the total energy of the relaxed water–cluster complex, *E*(cluster) is the total energy of the isolated cluster in its optimized geometry, and *E*(H_2_O) is the total energy of an isolated H_2_O molecule at its optimized geometry. All total energies include zero-point energy corrections. Under this convention, negative values of *E*_a_ correspond to exothermic (thermodynamically favorable) adsorption, while positive values indicate endothermic (unfavorable) adsorption. All total energies used in these calculations are provided in the Supplementary Materials to ensure full reproducibility.

Choice of the H_2_O^−^ reference state. In this study, all clusters were treated as negatively charged species (total charge −1) because the red shifts of the *ν*_1_/*ν*_3_ center for H_2_O adsorbed on negatively charged single atoms are considerably larger than those for neutral or positively charged species, as shown in Figure 1. To maintain internal consistency, the vibrational reference state was chosen as the negatively charged isolated water molecule (H_2_O^−^) rather than neutral H_2_O, ensuring that both the reference and the adsorbed systems share the same charge state. This choice eliminates charge-state effects from the vibrational comparison and isolates the perturbation induced by cluster adsorption on the stretching frequencies.

The gas-phase H_2_O^−^ anion has been established as a bound species in high-level ab initio calculations, with the excess electron stabilized by ion–quadrupole interactions (binding energy ~0.2 eV relative to H_2_O + e^−^) [48]. Although the vertical electron affinity of water in the Franck–Condon region is extremely small (~10^−4^ eV), the anion is computationally tractable with the present method. The DNP basis set used in this study includes diffuse functions, which are essential for describing the weakly bound anionic state of H_2_O^−^. This basis set choice ensures reliable geometries and frequencies for the reference state.

The vibrational descriptor Δ*IR* is defined as the red shift in the intensity-weighted center of the coupled *ν*_1_ (symmetric stretching) and *ν*_3_ (antisymmetric stretching) modes of adsorbed H_2_O relative to the corresponding frequency of negatively charged isolated H_2_O. Specifically, Δ*IR* = *ν*_ref_ − *ν*_adsorbed_, where $\nu_{adsorbed}=\frac{I_{1}^{ads}v_{1}^{ads}+\;I_{3}^{ads}v_{3}^{ads}}{I_{1}^{ads}+\;I_{3}^{ads}}$, and $\nu_{ref}=\frac{I_{1}^{ref}v_{1}^{ref}+\;I_{3}^{ref}v_{3}^{ref}}{I_{1}^{ref}+\;I_{3}^{ref}}$. Here, *ν_i_* and *I_i_* denote the harmonic vibrational frequency and infrared intensity of mode *i*, respectively. The subscript “ads” refers to H_2_O adsorbed on the cluster, and “ref” refers to the negatively charged isolated H_2_O molecule (H_2_O^−^). Under this sign convention, a positive Δ*IR* value corresponds to a red shift (i.e., the adsorbed frequencies are lower than the reference), while a negative value corresponds to a blue shift. All vibrational frequencies were calculated at the optimized geometry of each system using the PBE functional and were applied consistently to all reported frequencies.

To investigate the subsequent dissociation dynamics, first-principles molecular dynamics (FPMD) simulations were performed using the NVE ensemble. The temperature range investigated was 50–1500 K. For all systems, simulations were conducted at 50 K and then from 100 K to 1500 K with a step size of 100 K. The lower bound of 50 K was included to capture systems that dissociate near cryogenic temperatures. All simulations were initiated from the optimized adsorbed (precursor) geometries of H_2_O on each cluster. The exothermic adsorption event itself—i.e., the release of adsorption energy upon H_2_O approaching the cluster—was not directly simulated. Rather, the simulations probe the thermal dissociation of the pre-adsorbed water molecule. The time step was set to 1 fs, and the total simulation time was 5 ps per trajectory. The O–H bond length was monitored throughout each trajectory, with dissociation defined as occurring when an O–H distance exceeded 1.5 Å (approximately twice the equilibrium O–H bond length in adsorbed H_2_O) and remained broken for the remainder of the 5 ps trajectory. Systems in which the H atom recombined with OH within the simulation time were classified as non-dissociative at that temperature.

We note that the dissociation temperature reported here is defined as the lowest initial temperature at which O–H bond cleavage was observed during a 5 ps NVE trajectory. Due to the computational cost of simulating 88 clusters, single trajectories were performed at each temperature. Consequently, the reported temperatures should be understood as qualitative indicators of catalytic activity rather than as statistically converged kinetic thresholds. The dependence on initial velocity distribution, trajectory duration, and temperature fluctuations in small isolated systems introduces uncertainty that should be considered when interpreting precise numerical values.

We constructed 88 clusters (Table 1) initially. For the selected systems in Table 2, three independent trajectories were performed with different initial velocity distributions to assess statistical variability. The dissociation temperature *T*_d_ for these systems is defined as the lowest temperature at which at least two of the three trajectories exhibited dissociation. For the remaining systems, single-trajectory simulations were conducted due to computational cost; these results are intended as qualitative indicators of relative catalytic activity rather than precise kinetic thresholds. All relevant computational details are provided in the Supplementary Materials.

## 3. Results and Discussion

### 3.1. Identification and Construction of Catalytic Systems

We first investigated the adsorption behavior of H_2_O on the first 75 single atoms of the periodic table, considering three charge states: positive, neutral, and negative (Figure 1). The red shifts of the center of the *ν*_1_ and *ν*_3_ modes of adsorbed H_2_O were calculated relative to isolated electrically neutral H_2_O. Notably, the red shift for H_2_O adsorbed on negatively charged single atoms is considerably larger than that for the other two charge states, exhibiting a periodic dependence on the elemental nature. Interestingly, inert elements in the negative charge state display high activity because their high stability facilitates electron transfer to water molecules, thereby rendering the water molecules more activated. Consequently, the extent of the red shift in the *ν*_1_/*ν*_3_ center for H_2_O adsorbed on inert elements is comparable to that observed for directly negatively charged water molecules.

Catalytic water dissociation is typically achieved using Cu and Pt metal surfaces [9,38]. The red shift in the *ν*_1_/*ν*_3_ center for H_2_O adsorbed on the negatively charged Cu atom is 615 cm^−1^, as indicated by the green dash-dotted line in Figure 1. Based on this reference, we constructed 24 diatomic, 30 triatomic, and 34 tetraatomic alloy clusters from multiple initial configurations, selecting the lowest-energy optimized structure for each composition, primarily incorporating Pt, along with alkali metals (Na) and elements exhibiting red shifts exceeding that of copper (Table 1).

Of the 88 clusters initially constructed (Table 1), 84 were successfully optimized and used for subsequent vibrational frequency calculations and FPMD simulations. The remaining four systems (NaPt-H_2_O, CdPt-H_2_O, Ni_2_Pt-H_2_O, and Ni_3_-H_2_O) failed to converge during geometry optimization and were therefore excluded from all reported analyses. A complete summary of all 88 clusters, including convergence status and calculated properties, is provided in Supplementary Materials.

**Table 1:** List of 24 diatomic, 30 triatomic, and 34 tetraatomic clusters investigated in this study.

Diatomic clusters 24	BePt	CPt	NPt	FPt	NaPt	PPt
	NiPt	Cu_2_	CuPt	AsPt	SrCd	SrRe
	SrPt	TcPt	PdPt	Cd_2_	CdRe	CdPt
	SbPt	Re_2_	RePt	IrPt	Pt_2_	PtAu
Triatomic clusters 30	Be_2_Pt	C_2_Pt	N_2_Pt	F_2_Pt	Na_2_Pt	NaPt_2_
	P_2_Pt	Ni_3_	Ni_2_Pt	Cu_3_	Cu_2_Pt	As_2_Pt
	Sr_2_Cd	Sr_2_Re	Sr_2_Pt	SrCd_2_	SrRe_2_	SrPt_2_
	Tc_2_Pt	Pd_2_Pt	Cd_3_	Cd_2_Re	Cd_2_Pt	CdRe_2_
	Sb_2_Pt	Re_3_	Re_2_Pt	Ir_2_Pt	Pt_3_	PtAu_2_
Tetraatomic clusters 34	Be_3_Pt	C_3_Pt	N_3_Pt	F_3_Pt	Na_4_	Na_3_Pt
	Na_2_Pt_2_	NaPt_3_	P_3_Pt	Ni_3_Pt	NiPt_3_	Cu_4_
	Cu_3_Pt	As_3_Pt	Sr_4_	Sr_3_Cd	Sr_3_Re	Sr_3_Pt
	Sr_2_Re_2_	Sr_2_Pt_2_	SrCd_3_	SrRe_3_	SrPt_3_	Tc_3_Pt
	Pd_3_Pt	Cd_4_	Cd_3_Re	Cd_3_Pt	CdRe_3_	Sb_3_Pt
	Re_4_	Re_3_Pt	Ir_3_Pt	Pt_4_		

### 3.2. Dependence of Dissociation Temperature on Infrared Vibrational Spectra

**Figure 2:**
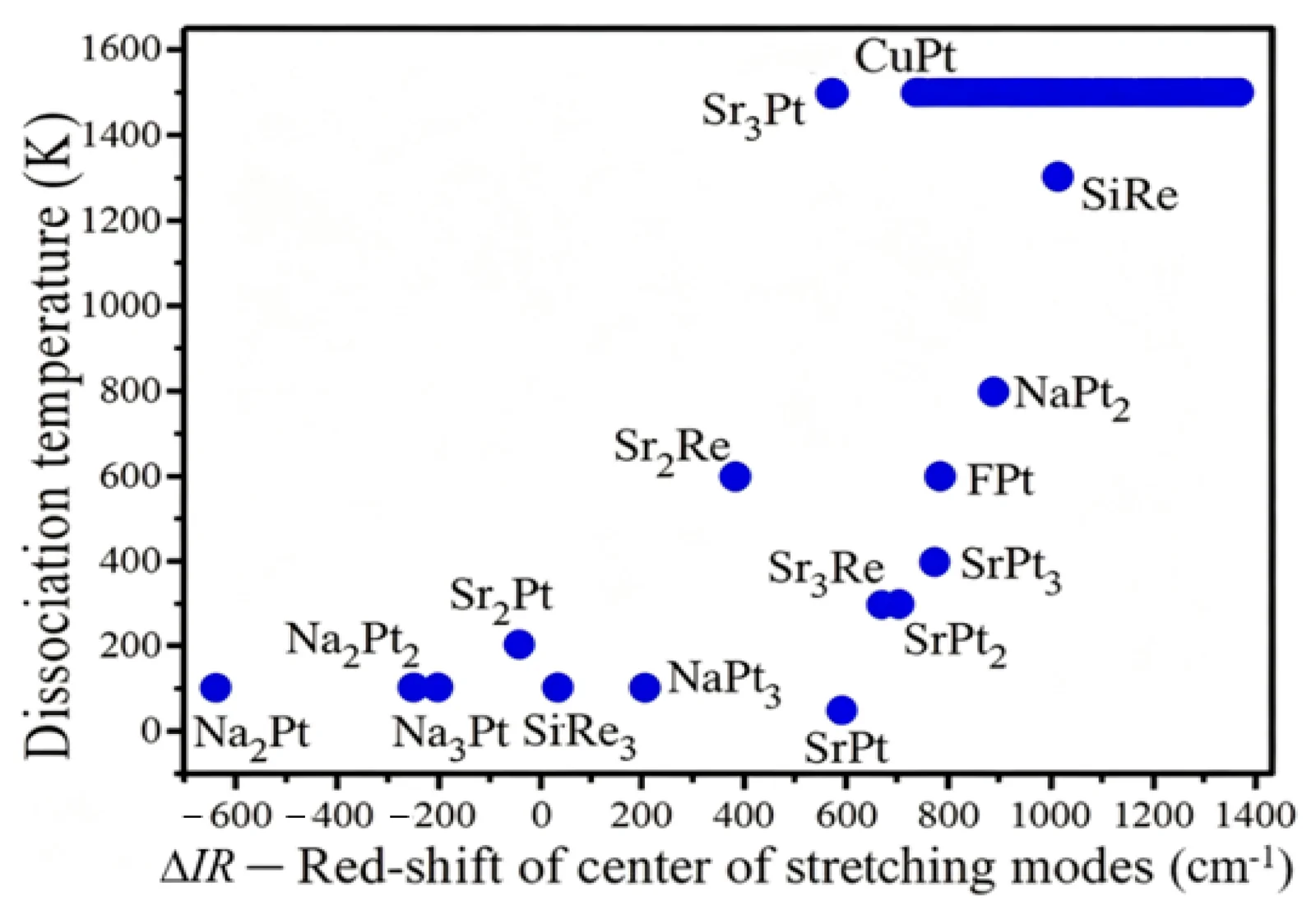
Scatter plot showing the relationship between the catalytic dissociation temperature (*T*_d_) and the vibrational descriptor Δ*IR* for H_2_O adsorbed on various clusters. Each blue circle represents an individual cluster. Clusters with *T*_d_ > 1500 K are plotted at 1500 K for convenience.

For catalysts that did not exhibit dissociation within the investigated temperature range (50–1500 K), the dissociation temperature is reported as “>1500 K”. In Figure 2, these systems are marked at 1500 K for plotting convenience, but it should be understood that this is a lower bound and not an exact numerical value. These observations constitute right-censored data and are treated accordingly in the statistical analysis. Clusters with Δ*IR* less than 300 cm^−1^ exhibit high catalytic activity for water dissociation, corresponding to dissociation temperatures not exceeding 200 K. Clusters with Δ*IR* values between 300 and 700 cm^−1^, with the exception of Sr_3_Pt and CuPt, still demonstrate high catalytic activity, with dissociation temperatures not higher than 600 K. In the Δ*IR* range of 700–1016 cm^−1^, significant variations in catalytic activity are observed among different catalysts. Catalysts corresponding to larger Δ*IR* values show no appreciable catalytic activity. These results demonstrate that the red shift in the *ν*_1_/*ν*_3_ center, which accounts for the strong coupling between the two stretching modes [26,27], serves as an effective descriptor for characterizing the catalytic reactivity of water molecules.

Previous theoretical studies have shown that water dissociation on metal surfaces typically proceeds through a late-barrier reaction and that vibrational excitation of the stretching modes can significantly enhance reactivity in accordance with Polanyi’s rules and the sudden vector projection (SVP) model [37,38,39,40]. Our observed correlation between the red shift in the stretching-mode center and the dissociation temperature is qualitatively consistent with this established picture. However, we note that a definitive determination of the barrier location for the present cluster systems would require explicit transition-state calculations, which are beyond the scope of this study.

While the red shift in the stretching-mode center is an effective descriptor of catalytic reactivity, the origin of certain exceptions remains unclear. Multiple factors, including molecular orbitals near the Fermi level, tunneling effects, resonances, zero-point energy (ZPE) effects, and electron spin polarization, can influence water reactivity [36,37,38,49,50]. In the following section, we examine the mechanism of catalytic water activation in greater detail.

### 3.3. Energy Matching Between Adsorption Energy and Homo–Lumo Gap

Faradzhev et al. demonstrated that water dissociation on Ru(0001) can be achieved through either thermal or electronic excitation [51]. To elucidate the underlying mechanisms, we considered charge transfer, adsorption energy, bond length, frontier orbitals, and density of states. To examine the relationship between the heat of adsorption and electronic excitation, we analyzed the LUMO–HOMO gap (*E*_g_) and the adsorption energy of H_2_O (*E*_a_). These parameters for catalysts with high catalytic activity and several inactive catalysts, along with the corresponding dissociation temperatures, are listed in Table 2.

**Table 2:** Selected catalytic descriptors for H_2_O dissociation on various clusters. *E*_g_ is the Kohn–Sham HOMO–LUMO gap; *E*_a_ is the adsorption energy of H_2_O defined as *E*_a_ = *E*(H_2_O/cluster) − *E*(cluster) − *E*(H_2_O) (negative values indicate exothermic adsorption); and *T*_d_ is the dissociation temperature determined from FPMD simulations (see Section 2 for details). Systems marked as >1500 K did not exhibit dissociation within the investigated temperature range (50–1500 K). These values represent lower bounds and are treated as right-censored data in statistical analyses.

Systems	∆*IR* (1/cm)	*E*_g_ (eV)	*E*_a_ (eV)	*E*_a_ + *E*_g_ (eV)	*T*_d_ (K)
Na_2_Pt	−640.5	0.71	−0.90	−0.19	100
Na_2_Pt_2_	−249.5	0.75	−0.97	−0.22	100
Na_3_Pt	−203.1	0.67	−0.86	−0.19	100
Sr_2_Pt	−42.8	0.60	−0.80	−0.20	200
SrRe_3_	34.1	0.28	−0.58	−0.30	100
Sr_2_Re	381.3	0.73	−0.68	0.05	600
CuPt	578.5	0.35	−1.02	−0.67	1500
SrPt	591.8	0.74	−0.76	−0.02	50
Sr_3_Re	673.8	0.59	−0.68	−0.09	300
SrPt_2_	702.0	0.96	−1.09	−0.13	300
Sr_2_Pt_2_	745.3	0.45	0.59	1.04	1500
SrPt_3_	774.9	0.73	−0.98	−0.25	400
FPt	787.4	2.95	−0.84	2.11	600
NaPt_2_	892.9	0.44	−1.01	−0.57	800
SrRe	1015.9	0.55	0.30	0.85	1300

From the data presented in Table 2, an empirical pattern emerges: lower dissociation temperatures tend to occur when the sum of the HOMO–LUMO gap and the adsorption energy (*E*_g_ + *E*_a_) is close to zero. To quantify this relationship, we performed a statistical correlation analysis. Following a predefined outlier exclusion criterion—systems with |*E*_g_ + *E*_a_| deviating by more than 3 standard deviations (3σ) from the mean of the dataset were classified as outliers—two systems (SrRe_3_ and FPt) were excluded. Before outlier exclusion, the Pearson correlation coefficient between *T*_d_ and |E_g_ + E_a_| was *R* = 0.78. After excluding the two outliers, the Pearson correlation coefficient improved to *R* = 0.90. These results indicate that when the energy released upon H_2_O adsorption is comparable to the HOMO–LUMO gap, the catalytic dissociation reaction tends to occur more readily. Conversely, when |*E*_g_ + *E*_a_| exceeds 0.25 eV, the reaction is generally unfavorable.

**Figure 3:**
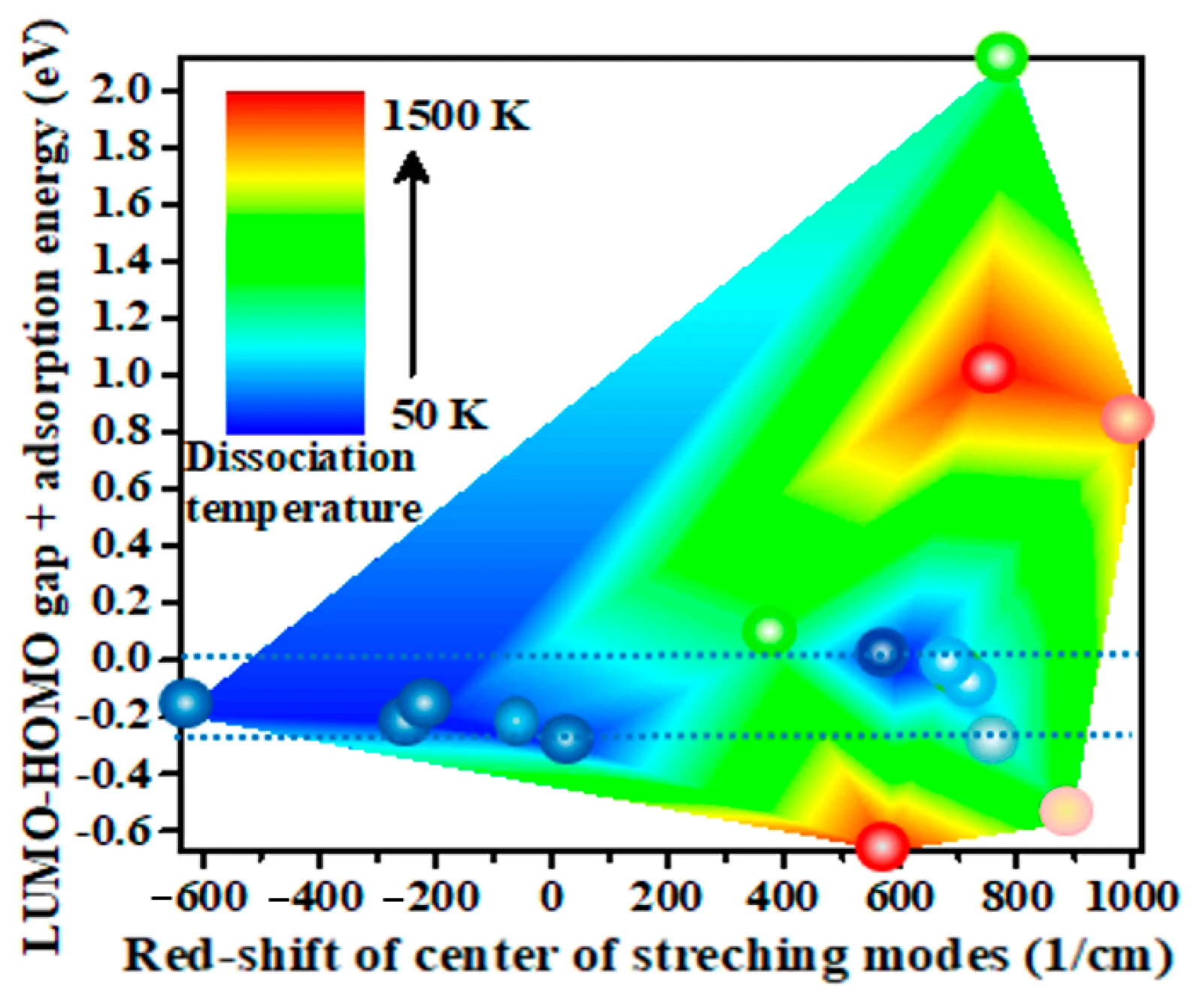
Three-dimensional correlation between the dissociation temperature (*T*_d_), the electronic descriptor (*E*_g_ + *E*_a_), and the vibrational descriptor (Δ*IR*). The color of each sphere indicates the dissociation temperature. Δ*IR* is defined as Δ*IR* = *ν*_ref_ − *ν*_adsorbed_, where the reference is negatively charged isolated H_2_O (H_2_O^−^). The blue dashed lines mark the region where |*E*_g_ + *E*_a_| ≤ 0.30 eV.

The observed correlation between |*E*_g_ + *E*_a_| and *T*_d_ suggests that energy matching between the adsorption energy and the HOMO–LUMO gap may facilitate the dissociation reaction, as shown in Figure 3. We tentatively interpret this as evidence for a resonance-like electronic effect. However, we emphasize that this interpretation is based on correlations with equilibrium ground-state properties rather than direct simulations of energy transfer dynamics. Explicit demonstration of a resonant energy-transfer mechanism would require nonadiabatic or excited-state dynamics calculations, which are beyond the scope of the present work.

Although the adsorption energy of water on Sr_2_Re is 0.05 eV lower than the HOMO–LUMO gap, we tentatively attribute the observed reactivity to the combined effects of near-resonant electronic conditions and the participation of multiple frontier orbitals in the reaction (see Section 3.4). We acknowledge that alternative interpretations—such as possible quantum effects—cannot be assessed within the present classical-nuclei Born–Oppenheimer framework and would require dedicated path-integral or quantum dynamics simulations.

For FPt, *E*_g_ + *E*_a_ = 2.11 eV, far from the resonance region, yet the dissociation temperature is 600 K. We hypothesize that this anomalous behavior may be related to the specific dissociation pathway: t + he products are H adsorbed on the Pt atom and a free HO group, which could potentially lead to a different entropy change compared to other systems. However, we emphasize that this entropy-based interpretation is speculative, as we have not performed quantitative free-energy calculations. A more rigorous analysis of the free-energy landscape would be required to confirm this hypothesis.

Overall, we emphasize that the mechanistic interpretations presented above—including the roles of electronic resonance, frontier orbital participation, and possible entropic effects—are suggested by the observed correlations but should be regarded as tentative hypotheses. Direct verification of these mechanisms would require dedicated nonadiabatic dynamics simulations, transition-state calculations, and/or free-energy analyses, which are beyond the scope of the present work.

It is important to clarify what the present calculations do and do not show regarding the role of adsorption energy. Our FPMD simulations begin from the optimized adsorbed state; therefore, the release of adsorption energy upon H_2_O approaching the cluster surface—and its subsequent transfer to vibrational or electronic degrees of freedom—is not directly captured in the dynamics. The relationship between *E*_a_ and *T*_d_ reported here is based on equilibrium total energy differences, not on explicit simulations of energy redistribution during the adsorption event. Direct investigation of energy-transfer pathways would require methods beyond ground-state Born–Oppenheimer dynamics, such as nonadiabatic molecular dynamics or time-dependent DFT. This limitation should be borne in mind when interpreting the proposed resonance effect.

### 3.4. Role of Frontier Molecular Orbitals (FMOs)

The concept of locality in physical space is fundamental to understanding chemical reactivity. Frontier molecular orbitals (FMOs) identify the chemically reactive regions based on associated orbital energies and have proven invaluable for describing chemical reactivity [52,53]. To gain deeper insight into the reaction kinetics of water dissociation, we performed a detailed analysis of the frontier orbitals of catalysts with adsorbed H_2_O molecules and those of the intermediate states in the catalytic dissociation reaction (Figure S1). By comparing the FMOs of reaction precursors and intermediate states, we identified which orbitals participate in the dissociation process.

The configuration of the frontier orbitals (HOMO-1, HOMO, and LUMO) of precursors is largely identical to that of the intermediate states, indicating that the FMOs of reaction precursors play a pivotal role in the catalytic process. In a few instances, the orbital energy ordering undergoes slight alterations. For example, as shown in Figure S1, the HOMO and LUMO of SrRe–H_2_O (precursor) exhibit a reversal, while the HOMO-1 and HOMO of Sr_2_Re–H_2_O (precursor) shift upward to become the HOMO and LUMO of the intermediate state, respectively.

Comparison of the frontier orbitals (HOMO-1, HOMO, and LUMO) of the precursor and intermediate state reveals that the number of orbitals involved in the reaction—defined as orbitals from the catalyst that exhibit increased overlap with H atoms during H_2_O dissociation—is inversely proportional to the corresponding dissociation temperature. To support this qualitative visual assessment, we performed Mulliken population analysis on the H atom in the precursor and intermediate states. For systems with multiple frontier orbitals involved, the change in Mulliken charge on the dissociating H atom between precursor states is larger (e.g., 0.106 e for SrRe_3_ vs. 0.182 e for CuPt), consistent with stronger catalyst–H interaction when more frontier orbitals participate. All orbital isosurface plots used a consistent value of 0.03 e/Å^3^.

This observation is qualitatively consistent with the expectation that greater frontier-orbital participation corresponds to stronger interaction between the dissociating H atom and the catalytic cluster, which may facilitate the cleavage of the O–H bond. This phenomenon has been observed by Kawai and coworkers in their study of the role of molecular orbitals near the Fermi level in the excitation of vibrational modes of a single water molecule, where chemisorption onto a metal surface leads to molecule–metal bond formation and orbital rehybridization [54].

**Figure 4:**
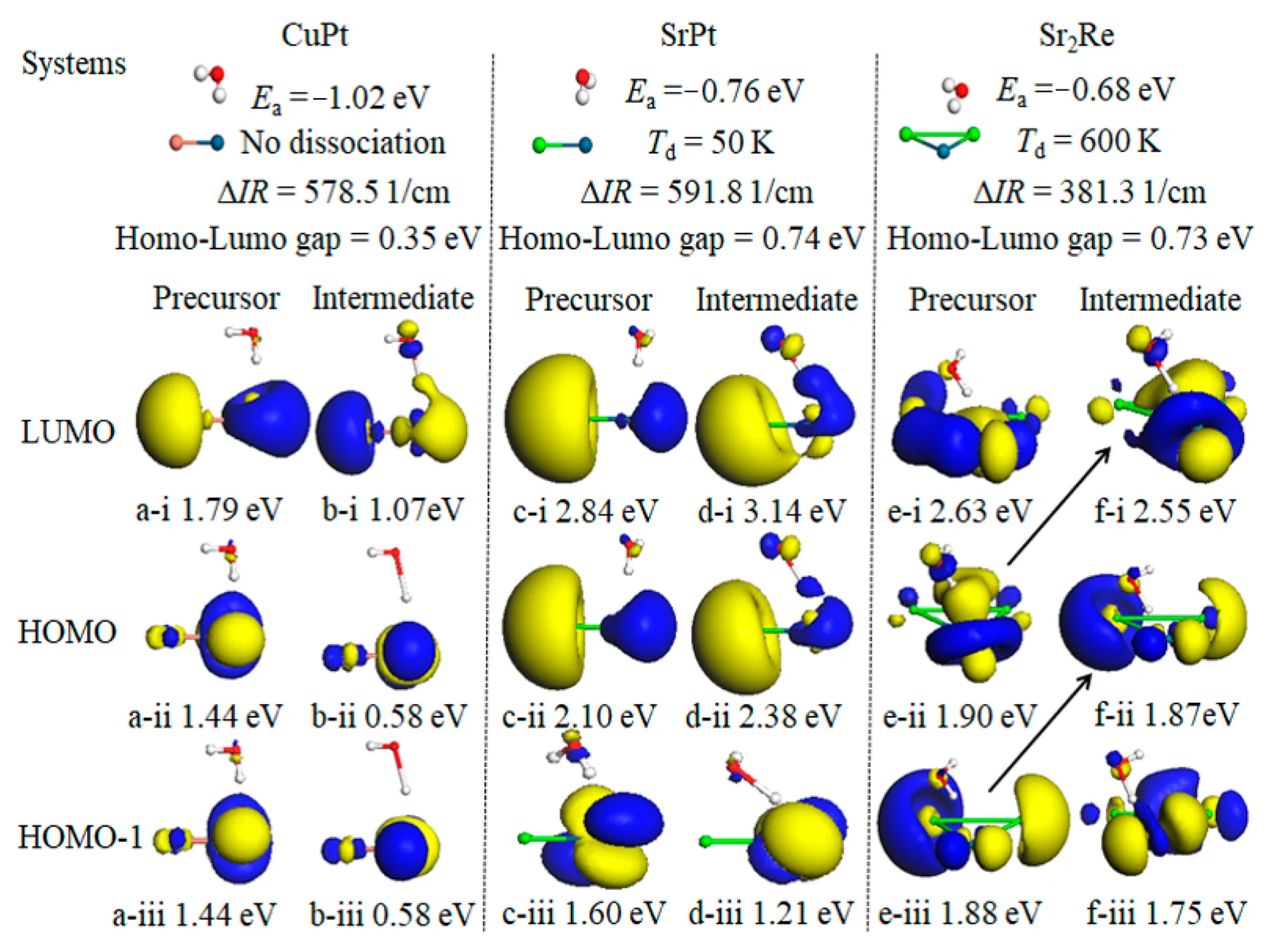
Frontier orbitals (HOMO-1, HOMO, and LUMO) of precursors and intermediate states in the dissociation reaction for CuPt, SrPt, and Sr_2_Re. *E*_a_: adsorption energy of H_2_O; Δ*IR*: red shift in *ν*_1_/*ν*_3_ center; *T*_d_: dissociation temperature.

In Figure 4, we use CuPt, SrPt, and Sr_2_Re as representative examples to illustrate the influence of frontier orbitals on catalytic water dissociation. For CuPt, comparison of the two sets of frontier orbitals (left panel) reveals that only the LUMO exhibits increased overlap with the dissociating H atom during the reaction, without the assistance of energy matching. Consequently, this material cannot effectively catalyze water dissociation below 1500 K. From an energetic perspective, the HOMO–LUMO gap (*E*_g_) and the energy released upon water adsorption are nearly identical for SrPt, satisfying the prerequisite for energy matching. Furthermore, both the HOMO and LUMO orbitals are involved in the H-dissociation process, as observed in the frontier orbitals of the intermediate state. Both factors contribute to the lower dissociation temperature on SrPt. For Sr_2_Re (right panels), the HOMO-1 and LUMO orbitals of the intermediate state participate in the catalytic process through a change in orbital energy ordering, thereby lowering the dissociation temperature compared to CuPt. Similarly, for SrRe_3_ (Figure S1e), the HOMO-1, HOMO, and LUMO orbitals are all involved in the catalytic dissociation reaction, resulting in a dissociation temperature as low as 100 K even under conditions away from energy resonance.

Several limitations should be noted: (i) the calculations are restricted to isolated gas-phase clusters without supports, solvents, or electrode potentials; (ii) the dissociation temperatures are qualitative indicators from single trajectories; and (iii) the proposed resonance mechanism requires validation by nonadiabatic dynamics. These factors should be considered when relating our findings to practical catalysis.

## 4. Conclusions

In summary, the vibrational descriptor Δ*IR*—defined as the intensity-weighted center of the coupled *ν*_1_ and *ν*_3_ stretching modes of adsorbed H_2_O relative to that of negatively charged isolated H_2_O (Δ*IR* = *ν*_ref_ − *ν*_adsorbed_)—serves as a useful spectroscopic descriptor for evaluating the catalytic activation of water molecules. The consistency of this definition across all figures, tables, and discussions ensures the reliability and reproducibility of our findings. However, a comprehensive understanding of the activation mechanism requires consideration of additional factors, including the degree of frontier orbital participation and entropic effects.

Notably, our results suggest a correlation between the catalytic activity and the proximity of the adsorption energy to the HOMO–LUMO gap, which we tentatively interpret as evidence for a resonance-like electronic effect. The number of frontier orbitals exhibiting increased overlap with the dissociating H atom also shows an inverse correlation with the dissociation temperature. While these observations provide useful insights into possible activation mechanisms, we emphasize that direct confirmation of the proposed electronic resonance mechanism would require nonadiabatic or excited-state calculations beyond the present ground-state DFT framework.

These findings provide a theoretical foundation for understanding water dissociation on model alloy clusters and suggest spectroscopic descriptors that may guide future catalyst design. We anticipate that our results will stimulate further experimental and theoretical investigations, particularly those incorporating support effects, solvation, and realistic reaction conditions.

We note that the present calculations were performed on unsupported gas-phase anionic clusters as model systems; extension to supported catalysts under realistic conditions requires further investigation.

## Data Availability

The complete dataset for all 88 clusters is available in the Supplementary Materials.

## Supplementary Materials

The following supporting information can be downloaded at https://www.mdpi.com/article/10.3390/molecules31183165/s1.
