# Supplementary material for: Correlations Between Vibrational Red Shifts, Electronic Structure, and Water Dissociation on Alloy Clusters: An Exploratory Computational Study
Source: Molecules. 2026 Sep 8;31(18):3165. doi: 10.3390/molecules31183165 (PMC13609100; doi:10.3390/molecules31183165)
Supplement: Supplementary file 1 [file molecules-31-03165-s001.zip › molecules-4468257-supplementary.pdf]

Materials and Methods

Methods

All calculations are implemented in the DMol3 package. The structural optimization of catalyst and determination of infrared spectrum of water adsorbed were carried out in the scheme of Density Functional Theory with Generalized Gradient Approximation (GGA) and Perdew–Burke–Ernzerhof (PBE) functional and a DFT–based relativistic semi-core pseudo potential (DSPP). The details are presented below.

#Optimize
Opt_energy_convergence 1.0000e-005
Opt_gradient_convergence 2.0000e-003 Å
Opt_displacement_convergence 5.0000e-003 Å
Opt_max_displacement 0.1000 Å
Scf_density_convergence 1.000000e-006
Scf_charge_mixing 2.000000e-001
Scf_spin_mixing 5.000000e-001
Scf_diis 8 pulay
Electronic parameters
Spin_polarization unrestricted
Charge -1
Basis dnp
Pseudopotential dspp
Functional pbe
Aux_density octupole
Integration_grid fine
Occupation fermi
Cutoff_Global 4.5000 angstrom

The catalytic dissociation process of H_2O molecules was simulated in the NVE ensemble with a temperature step of 100K in the temperature range of 0 to 1500K. The details are listed below.

#Molecular_dynamics
Write_HIS_File on
Write_ARC_File on
MD_Velocity random
MD_Time_Step 1.0000
MD_Simann_panel
5000 MD_NVE 100.0000
Symmetry off
Scf_density_convergence 1.000000e-006
Scf_charge_mixing 2.000000e-001
Scf_spin_mixing 5.000000e-001
Scf_diis 8 pulay
Electronic parameters
Spin_polarization unrestricted
Charge -1
Basis dnp
Pseudopotential dspp
Functional pbe
Aux_density octupole
Integration_grid fine
Occupation fermi

Cutoff_Global 4.5000 angstrom

Supplementary Text

The coordinates of catalysts (in Table 1) adsorbing H_2O (reaction precursors) along with the total energy and vibrational frequencies of H_2O, some intermediate states are represented below.

H_2O (Uncharged)

Final Coordinates (Angstroms)

	ATOM	X	Y	Z
1	O	0.050478	0.000000	0.050478
2	H	1.015226	0.000000	-0.065704
3	H	-0.065704	0.000000	1.015226

Total Energy -76.4621905 Ha

Vibrational frequencies of H_2O, intensities

Mode	au_amu	cm-1	km/mol
7	0.316363	1626.3	74.85
8	0.722145	3712.2	4.92
9	0.744670	3828.0	44.43

H_2O^- (Negatively charged)

Final Coordinates (Angstroms)

	ATOM	X	Y	Z
1	O	0.013371	0.000000	0.013371
2	H	1.050655	0.000000	-0.064026
3	H	-0.064026	0.000000	1.050655

Total Energy -76.3520460 Ha

Vibrational frequencies of H_2O, intensities

Mode	au_amu	cm-1	km/mol
7	0.235613	1211.2	85.26
8	0.378006	1943.1	4406.84
9	0.517973	2662.6	2.59

1.1 $BePt^-$

Final Coordinates (Angstroms)

	ATOM	X	Y	Z
1	Be	0.082655	-0.580791	0.187043
2	Pt	2.049022	-0.451264	0.121255

Total Energy -209.9005987 Ha

1.2 $BePtH_2O^-$

Final Coordinates (Angstroms)

ATOM		X	Y	Z
1	Be	0.065326	-0.581933	0.187623
2	Pt	2.066351	-0.450123	0.120675
3	H	0.127609	2.358709	-0.914546
4	H	1.496292	1.781947	-0.652572
5	O	1.060951	2.629873	-0.966995

Total Energy -286.6318121 Ha

Vibrational frequencies of H_2O, intensities

Mode	au_amu	cm-1	km/mol
13	0.321042	1650.3	187.98
14	0.612911	3150.7	878.99
15	0.727550	3740.0	4.02

2.1 CPt^-

Final Coordinates (Angstroms)

ATOM		X	Y	Z
1	C	0.395874	-0.107547	0.627403
2	Pt	1.917812	-0.689414	-0.090596

Total Energy -233.1940656 Ha

2.2 $CPtH_2O^-$

Final Coordinates (Angstroms)

ATOM		X	Y	Z
1	C	0.136337	-0.405331	0.113332
2	Pt	1.905820	-0.554706	0.152025
3	H	0.195540	2.017685	-0.748013
4	H	1.663128	2.036411	-0.769168
5	O	0.915705	2.644414	-0.973992

Total Energy -309.9147842 Ha

Vibrational frequencies of H_2O, intensities

Mode	au_amu	cm-1	km/mol
13	0.324086	1666.0	282.28
14	0.685124	3521.9	263.92
15	0.699504	3595.8	97.77

3.1 NPt^-

Final Coordinates (Angstroms)

ATOM		X	Y	Z
1	N	0.150549	-0.315716	0.065825
2	Pt	1.922868	-0.558971	0.170829

Total Energy -249.8904023 Ha

3.2 $NPtH_2O^-$

Final Coordinates (Angstroms)

ATOM		X	Y	Z
1	N	0.147946	-0.315359	0.065671
2	Pt	1.925471	-0.559329	0.170983
3	H	0.181901	2.002170	-0.737938
4	H	1.650366	1.997321	-0.760075
5	O	0.910845	2.613670	-0.964455

Total Energy -326.6074450 Ha

Vibrational frequencies of H_2O, intensities

Mode	au_amu	cm-1	km/mol
13	0.325367	1672.5	311.24
14	0.688790	3540.7	264.73
15	0.713175	3666.1	31.20

4.1 FPt^-

Final Coordinates (Angstroms)

ATOM		X	Y	Z
1	F	0.066007	-0.232255	0.059616
2	Pt	2.014784	-0.445243	0.109337

Total Energy -295.2703729 Ha

4.2 $FPtH_2O^-$

Reaction precursor

Final Coordinates (Angstroms)

ATOM		X	Y	Z
1	F	0.064684	-0.232110	0.059582
2	Pt	2.016107	-0.445387	0.109370
3	H	0.176147	1.946870	-0.721723
4	H	1.647378	1.919512	-0.730992
5	O	0.912213	2.549590	-0.942052

Total Energy -371.7635771 Ha

Vibrational frequencies of H_2O, intensities

Mode	au_amu	cm-1	km/mol
13	0.326232	1677.0	301.54
14	0.662907	3407.7	513.14
15	0.719880	3700.5	25.80

Intermediate state

$coordinates

F	6.22949742869010	1.82112117516002	1.49667412132631
Pt	3.31895319971326	-0.49079718608321	0.10963455873750
H	2.41495036240892	-3.28991462116834	0.20291989490187
H	1.66243724983933	-1.74376313485796	-3.50673551530046
O	0.41798644855777	-1.36597618576057	-2.20527917904057

5.1 $NaPt^-$

Final Coordinates (Angstroms)

	ATOM	X	Y	Z
1	Na	-0.292697	0.370240	-0.084431
2	Pt	2.129463	1.203879	-0.566264
3	H	0.056471	3.839610	-1.333161
4	H	1.451395	3.269463	-1.142574
5	O	0.976901	4.147801	-1.401211

Total Energy -434.151817Ha

5.2 $NaPtH_2O^-$

In this method, the structural optimization of $NaPtH_2O^-$ did not be completed successfully, because SCF iterations are always not converging.

6.1 PPt^-

Final Coordinates (Angstroms)

	ATOM	X	Y	Z
1	P	-0.056859	-0.361653	0.107586
2	Pt	2.085136	-0.359174	0.085619

Total Energy -536.4314704 Ha

6.2 $PPtH_2O^-$

Final Coordinates (Angstroms)

	ATOM	X	Y	Z
1	P	-0.040470	-0.822739	0.242896
2	Pt	2.064936	-0.435596	0.130280
3	H	0.164947	2.447333	-0.887541
4	H	1.528935	1.851397	-0.705847
5	O	1.098181	2.698078	-1.005603

Total Energy -613.2434896 Ha

Vibrational frequencies of H_2O, intensities

Mode	au_amu	cm-1	km/mol
13	0.323236	1661.6	152.80
14	0.643365	3307.2	804.94
15	0.729394	3749.4	7.06

7.1 $NiPt^-$

Final Coordinates (Angstroms)

	ATOM	X	Y	Z
1	Ni	-0.117471	-0.117419	0.029875
2	Pt	2.174941	-0.195056	0.017009

Total Energy -388.6322287 Ha

7.2 $NiPtH_2O^-$

Final Coordinates (Angstroms)

	ATOM	X	Y	Z
1	Ni	0.458099	0.158674	1.466470
2	Pt	1.987214	-1.175507	0.271026
3	H	1.084560	-1.078773	-3.032285
4	H	1.645001	-0.278091	-1.881303
5	O	1.458680	-0.196052	-2.869345

Total Energy -465.4249351 Ha

Vibrational frequencies of H_2O, intensities

Mode	au_amu	cm-1	km/mol
13	0.318719	1638.4	242.60
14	0.595218	3059.7	1242.78
15	0.730634	3755.8	6.73

8.1 Cu_2^-

Final Coordinates (Angstroms)

	ATOM	X	Y	Z
1	Cu	-0.210830	-0.488738	0.137430
2	Cu	2.192737	-0.485560	0.121626

Total Energy -444.8213986 Ha

8.2 $Cu_2H_2O^-$

Final Coordinates (Angstroms)

	ATOM	X	Y	Z
1	Cu	-0.241624	-0.488779	0.137633
2	Cu	2.223531	-0.485520	0.121424
3	H	0.198295	2.057730	-0.748389
4	H	1.681599	2.013736	-0.766424
5	O	0.954728	2.641307	-0.970059

Total Energy -521.6075393 Ha

Vibrational frequencies of H_2O, intensities

Mode au_amu cm-1 km/mol

13	0.321732	1653.9	296.25
14	0.693398	3564.4	292.99
15	0.700054	3598.6	18.23

9.1 $CuPt^-$

Final Coordinates (Angstroms)

	ATOM	X	Y	Z
1	Cu	-0.176949	-1.692620	0.516530
2	Pt	0.804737	0.398413	-0.158824

Total Energy -417.9463704 Ha

9.2 $CuPtH_2O^-$

Reaction precursor

Final Coordinates (Angstroms)

	ATOM	X	Y	Z
1	Cu	-0.176533	-0.929558	0.293107
2	Pt	2.102566	-0.271884	0.052016
3	H	0.199683	2.441403	-0.929510
4	H	1.551392	1.810867	-0.662390
5	O	1.139421	2.687645	-0.979038

Total Energy -494.4423177 Ha

Vibrational frequencies of H_2O, intensities

Mode	au_amu	cm-1	km/mol
13	0.316883	1628.9	143.48
14	0.557154	2864.0	1399.93
15	0.729431	3749.6	2.97

Intermediate state

$coordinates

Cu	-0.34170131160153	-1.75613902683959	0.55414510680965
Pt	3.98213773759133	-0.51417419995971	0.09850943112458
H	-0.51704331822295	5.83357490458279	-1.87072828307065
H	2.93003277419293	3.42090347062240	-1.25264315898617
O	1.25827109975765	6.30072193766512	-1.96788179034712

Total Energy -494.357712 Ha

10.1 $AsPt^-$

Final Coordinates (Angstroms)

	ATOM	X	Y	Z
1	As	-0.134711	-0.809930	0.182063
2	Pt	2.100022	-0.413106	0.177159

Total Energy -521.8115284 Ha

10.2 $AsPtH_2O^-$

Final Coordinates (Angstroms)

ATOM		X	Y	Z
1	As	-0.146075	-0.811948	0.182088
2	Pt	2.111385	-0.411088	0.177134
3	H	0.182212	2.410617	-0.890276
4	H	1.562060	1.861040	-0.690522
5	O	1.106947	2.689853	-1.004239

Total Energy -599.1299424 Ha

Vibrational frequencies of H_2O, intensities

Mode	au_amu	cm-1	km/mol
13	0.321936	1654.9	182.87
14	0.641367	3296.9	725.30
15	0.731051	3758.0	5.67

11.1 $SrCd^-$

Final Coordinates (Angstroms)

ATOM		X	Y	Z
1	Sr	-0.755492	-0.285416	0.071888
2	Cd	2.614660	0.054986	-0.070575

Total Energy -140.7225707 Ha

11.2 $SrCdH_2O^-$

Final Coordinates (Angstroms)

ATOM		X	Y	Z
1	Sr	-0.866091	-0.296587	0.076563
2	Cd	2.725258	0.066157	-0.075250
3	H	0.186590	2.957332	-1.256035
4	H	1.642053	2.637486	-1.125613
5	O	1.059626	3.384700	-1.431449

Total Energy -217.7065667 Ha

Vibrational frequencies of H_2O, intensities

Mode	au_amu	cm-1	km/mol
13	0.306994	1578.1	37.18
14	0.636879	3273.9	527.47
15	0.664906	3417.9	876.35

12.1 $SrRe^-$

Final Coordinates (Angstroms)

ATOM		X	Y	Z
1	Sr	-0.773685	-0.257651	0.096227
2	Re	2.566704	0.178827	-0.128788

Total Energy -202.6548507 Ha

12.2 $SrReH_2O^-$
Reaction precursor

Final Coordinates (Angstroms)

ATOM		X	Y	Z
1	Sr	-0.722270	-0.262407	0.092889
2	Re	2.519008	0.169803	-0.121581
3	H	0.203962	3.005579	-1.261107
4	H	1.630756	2.521278	-1.105347
5	O	1.115981	3.314835	-1.416638

Total Energy -279.1410990 Ha

Vibrational frequencies of H_2O, intensities

Mode	au_amu	cm-1	km/mol
13	0.315351	1621.1	381.09
14	0.637401	3276.6	572.31
15	0.718530	3693.6	39.35

Intermediate state
$coordinates

Sr	-1.34121306155084	0.07203744647728	-0.04943639950113
Re	4.91584188984126	0.24545826303373	-0.21527000346025
H	2.43297985386335	2.25367275585633	-1.38687610505845
H	0.78898186936058	5.18806392446407	-0.31371981183586
O	0.18371100080639	4.22089569130024	-1.78816600127104

Total Energy -279.144344 Ha

13.1 $SrPt^-$

Final Coordinates (Angstroms)

ATOM		X	Y	Z
1	Sr	-0.273730	-0.422980	0.308308
2	Pt	2.101097	0.325084	-0.433793

Total Energy -246.7832070 Ha

13.2 $SrPtH_2O^-$
Reaction precursor

Final Coordinates (Angstroms)

ATOM	X	Y	Z

1	Sr	-0.289455	-0.427933	0.313222
2	Pt	2.116822	0.330037	-0.438707
3	H	0.923064	3.133613	-1.983950
4	H	1.192810	2.421840	-0.671050
5	O	0.804195	3.291532	-1.031299

Total Energy -323.3032862 Ha

Vibrational frequencies of H_2O, intensities

Mode	au_amu	cm-1	km/mol
13	0.315521	1621.9	182.37
14	0.559103	2874.1	1145.10
15	0.727998	3742.3	6.76

Intermediate state
$coordinates

Sr	-0.78247401541351	-0.17284767963027	0.77132345775613
Pt	4.37458272532246	0.55463795305223	-1.01518763286849
H	-0.61763782969015	4.38293179045570	-2.17303766967132
H	3.04361541489118	2.93247071742569	-0.75227162068862
O	-1.65628527100585	3.78034613201828	-0.79349014362355

Total Energy -323.294287 Ha

14.1 $TcPt^-$

Final Coordinates (Angstroms)

	ATOM	X	Y	Z
1	Tc	-0.176774	-1.063910	0.351660
2	Pt	2.129457	-0.355381	0.046712

Total Energy -305.0142717 Ha

14.2 $TcPtH_2O^-$

Final Coordinates (Angstroms)

	ATOM	X	Y	Z
1	Tc	-0.190588	-1.068153	0.353487
2	Pt	2.143271	-0.351137	0.044885
3	H	0.216527	2.426482	-1.113468
4	H	1.568493	1.938331	-0.664328
5	O	1.078826	2.792951	-0.846392

Total Energy -381.8178808 Ha

Vibrational frequencies of H_2O, intensities

Mode	au_amu	cm-1	km/mol
13	0.321608	1653.2	181.13
14	0.620740	3190.9	886.73
15	0.726276	3733.4	6.85

15.1 $PdPt^-$

Final Coordinates (Angstroms)

ATOM		X	Y	Z
1	Pd	-0.239563	-0.837893	0.321934
2	Pt	2.173127	-0.303422	0.092422

Total Energy -358.4668688 Ha

15.2 $PdPtH_2O^-$

Final Coordinates (Angstroms)

ATOM		X	Y	Z
1	Pd	-0.263626	-0.843223	0.324223
2	Pt	2.197189	-0.298091	0.090133
3	H	0.189223	2.344647	-0.892490
4	H	1.597923	1.860015	-0.721208
5	O	1.095819	2.675126	-1.026473

Total Energy -435.2804158 Ha

Vibrational frequencies of H_2O, intensities

Mode	au_amu	cm-1	km/mol
13	0.322523	1657.9	201.47
14	0.607020	3120.4	895.67
15	0.726560	3734.9	1.60

16.1 Cd_2^-

Final Coordinates (Angstroms)

ATOM		X	Y	Z
1	Cd	-0.611203	0.021102	-0.042042
2	Cd	2.583810	0.035471	-0.061089

Total Energy -179.3611760 Ha

16.2 $Cd_2H_2O^-$

Final Coordinates (Angstroms)

ATOM		X	Y	Z
1	Cd	-0.731457	0.024680	-0.043947
2	Cd	2.701789	0.040026	-0.065580
3	H	0.172571	2.738813	-1.149587
4	H	1.654312	2.642881	-1.151414
5	O	0.950222	3.302688	-1.401255

Total Energy -256.1928849 Ha

Vibrational frequencies of H_2O, intensities

Mode	au_amu	cm-1	km/mol
13	0.309376	1590.3	27.51
14	0.625815	3217.0	799.56
15	0.647122	3326.5	1295.74

17.1 $CdRe^-$

Final Coordinates (Angstroms)

	ATOM	X	Y	Z
1	Cd	-0.535144	-1.607094	0.571205
2	Re	1.973247	-0.362026	0.071427

Total Energy -240.8268969 Ha

17.2 $CdReH_2O^-$

Final Coordinates (Angstroms)

	ATOM	X	Y	Z
1	Cd	-0.608529	-1.643520	0.585826
2	Re	2.046632	-0.325600	0.056805
3	H	0.352667	2.647617	-0.964290
4	H	1.755310	2.115247	-0.809926
5	O	1.270449	2.944730	-1.094231

Total Energy -317.6380089 Ha

Vibrational frequencies of H_2O, intensities

Mode	au_amu	cm-1	km/mol
13	0.317709	1633.2	265.22
14	0.614318	3157.9	1262.24
15	0.729677	3750.9	4.50

18.1 $CdPt^-$

In this method, the structural optimization of $CdPt^-$ did not be completed successfully, because SCF iterations are always not converging.

18.2 $CdPtH_2O^-$

In this method, the structural optimization of $CdPtH_2O^-$ did not be completed successfully, because SCF iterations are always not converging.

19.1 $SbPt^-$

Final Coordinates (Angstroms)

	ATOM	X	Y	Z
1	Sb	-0.207525	-1.035953	0.397405
2	Pt	2.149489	-0.356293	0.059055

Total Energy -336.0114771 Ha

19.2 $SbPtH_2O^-$

Final Coordinates (Angstroms)

ATOM		X	Y	Z
1	Sb	-0.225137	-1.041032	0.399933
2	Pt	2.167100	-0.351214	0.056527
3	H	0.202130	2.489153	-1.026268
4	H	1.533896	1.890541	-0.669358
5	O	1.138539	2.751025	-0.986649

Total Energy -412.8425485 Ha

Vibrational frequencies of H_2O, intensities

Mode	au_amu	cm-1	km/mol
13	0.322226	1656.4	148.52
14	0.629133	3234.1	930.13
15	0.729351	3749.2	4.98

20.1 Re_2^-

Final Coordinates (Angstroms)

ATOM		X	Y	Z
1	Re	-0.072452	-0.095435	-0.029864
2	Re	2.041423	0.067543	-0.026509

Total Energy -302.3417469 Ha

20.2 $Re_2H_2O^-$

Final Coordinates (Angstroms)

ATOM		X	Y	Z
1	Re	-0.079862	-0.096007	-0.029875
2	Re	2.048834	0.068115	-0.026497
3	H	0.173008	2.788226	-1.167636
4	H	1.649755	2.666799	-1.172093
5	O	0.955702	3.321956	-1.415682

Total Energy -379.1986907 Ha

Vibrational frequencies of H_2O, intensities

Mode	au_amu	cm-1	km/mol
13	0.322055	1655.5	322.43
14	0.680033	3495.7	299.36
15	0.706318	3630.8	52.79

21.1 $RePt^-$

Final Coordinates (Angstroms)

ATOM		X	Y	Z
1	Re	-0.212925	-0.776714	0.156534

2	Pt	2.146643	-0.393829	0.167404

Total Energy -346.4209389 Ha

21.2 RePtH_2O^-

Final Coordinates (Angstroms)

ATOM		X	Y	Z
1	Re	-0.228404	-0.779226	0.156463
2	Pt	2.162121	-0.391317	0.167475
3	H	0.186273	2.321297	-0.863876
4	H	1.615529	1.902437	-0.692669
5	O	1.081009	2.685283	-0.993208

Total Energy -423.2358819 Ha

Vibrational frequencies of H_2O, intensities

Mode	au_amu	cm-1	km/mol
13	0.323371	1662.3	233.17
14	0.647759	3329.8	553.16
15	0.724898	3726.3	14.11

22.1 $IrPt^-$

Final Coordinates (Angstroms)

ATOM		X	Y	Z
1	Ir	-0.204245	-0.544892	0.131911
2	Pt	2.193003	-0.453739	0.122252

Total Energy -373.0201024 Ha

22.2 IrPtH_2O^-

Final Coordinates (Angstroms)

ATOM		X	Y	Z
1	Ir	-0.224219	-0.545651	0.131991
2	Pt	2.212977	-0.452979	0.122171
3	H	0.186498	2.110588	-0.788178
4	H	1.657996	1.973101	-0.726965
5	O	0.983277	2.653415	-0.964835

Total Energy -449.8146886 Ha

Vibrational frequencies of H_2O, intensities

Mode	au_amu	cm-1	km/mol
13	0.324492	1668.0	276.39
14	0.671126	3449.9	268.21
15	0.699504	3595.8	66.43

23.1 Pt_2^-

Final Coordinates (Angstroms)

ATOM		X	Y	Z
1	Pt	-0.247970	-0.464370	0.120839
2	Pt	2.196939	-0.451188	0.112387

Total Energy -390.4859693 Ha

23.2 $Pt_2H_2O^-$

Final Coordinates (Angstroms)

ATOM		X	Y	Z
1	Pt	-0.265943	-0.464467	0.120901
2	Pt	2.214912	-0.451091	0.112325
3	H	0.215923	2.026554	-0.742410
4	H	1.689515	1.997845	-0.754955
5	O	0.962121	2.629632	-0.961676

Total Energy -467.2722981 Ha

Vibrational frequencies of H_2O, intensities

Mode	au_amu	cm-1	km/mol
13	0.323024	1660.5	311.11
14	0.681844	3505.0	233.76
15	0.686892	3531.0	83.94

24.1 $PtAu^-$

Final Coordinates (Angstroms)

ATOM		X	Y	Z
1	Au	-0.330205	-0.148905	-0.065178
2	Pt	2.210160	0.336774	-0.112600

Total Energy -406.6536832 Ha

24.2 $PtAuH_2O^-$

Final Coordinates (Angstroms)

ATOM		X	Y	Z
1	Au	-0.342068	-0.151173	-0.064956
2	Pt	2.222023	0.339042	-0.112822
3	H	0.181852	2.893265	-1.226723
4	H	1.601742	2.437511	-1.037137
5	O	1.083887	3.230444	-1.370146

Total Energy -483.6378140 Ha

Vibrational frequencies of H_2O, intensities

Mode	au_amu	cm-1	km/mol
13	0.320313	1646.6	191.85

14	0.609334	3132.3	897.29
15	0.729252	3748.7	4.81

25.1 Be_2Pt^-

Final Coordinates (Angstroms)

	ATOM	X	Y	Z
1	Pt	-0.029591	-0.019793	-0.036412
2	Be	2.071357	-0.060167	-0.110684
3	Be	1.074834	0.834457	1.535081

Total Energy -224.9091787 Ha

25.2 $Be_2PtH_2O^-$

Final Coordinates (Angstroms)

	ATOM	X	Y	Z
1	Pt	-0.131134	-0.094204	0.169845
2	Be	1.945487	-0.123112	-0.162465
3	Be	1.175338	0.432127	1.735945
4	H	0.333315	2.308851	-0.488083
5	H	1.614712	2.979793	-0.094335
6	O	0.792230	3.184407	-0.572758

Total Energy -301.3893185 Ha

Vibrational frequencies of H_2O, intensities

Mode	au_amu	cm-1	km/mol
16	0.321864	1654.5	240.72
17	0.653677	3360.2	425.51
18	0.726549	3734.8	5.45

26.1 C_2Pt^-

Final Coordinates (Angstroms)

	ATOM	X	Y	Z
1	C	0.171982	0.135486	0.249242
2	Pt	2.130112	-0.077332	-0.142260
3	C	0.814506	0.696342	1.281003

Total Energy -271.5163458 Ha

26.2 $C_2PtH_2O^-$

Final Coordinates (Angstroms)

	ATOM	X	Y	Z
1	Pt	-0.155979	-0.435130	0.282991
2	C	1.929243	-0.117485	0.335460
3	C	1.309208	0.174777	1.485676
4	H	0.203573	2.132559	-0.913030

5	H	1.670177	2.083869	-0.819020
6	O	0.976907	2.654380	-1.209907

Total Energy -347.9985376 Ha

Vibrational frequencies of H_2O, intensities

Mode	au_amu	cm-1	km/mol
16	0.326770	1679.8	265.27
17	0.705727	3627.8	239.20
18	0.711136	3655.6	5.73

27.1 N_2Pt^-

Final Coordinates (Angstroms)

	ATOM	X	Y	Z
1	N	0.667617	0.249581	0.459163
2	Pt	2.276680	-0.247914	-0.456294
3	N	0.172304	0.752829	1.385116

Total Energy -304.6821312 Ha

27.2 $N_2PtH_2O^-$

Final Coordinates (Angstroms)

	ATOM	X	Y	Z
1	N	-0.409261	1.388994	0.218868
2	Pt	2.432853	0.359356	0.184280
3	N	0.733269	1.275698	0.119375
4	O	1.316073	-2.580977	1.498580
5	H	1.794442	-1.849146	1.009697
6	H	0.633321	-2.059436	1.954159

Total Energy -381.4601022 Ha

Vibrational frequencies of H_2O, intensities

Mode	au_amu	cm-1	km/mol
15	0.319113	1640.4	184.87
17	0.619041	3182.2	1015.33
18	0.732201	3763.9	11.81

28.1 F_2Pt^-

Final Coordinates (Angstroms)

	ATOM	X	Y	Z
1	F	-0.037102	-0.541055	-0.995268
2	Pt	1.039525	0.251342	0.462300
3	F	2.114177	1.044210	1.920953

Total Energy -395.2250659 Ha

28.2 $F_2PtH_2O^-$

Final Coordinates (Angstroms)

	ATOM	X	Y	Z
1	Pt	0.802106	-0.169893	1.013774
2	F	1.908817	0.008225	-0.638337
3	F	-0.280949	-0.351165	2.646157
4	H	0.621603	2.734621	-0.904424
5	H	1.594366	1.682225	-1.356470
6	O	1.287188	2.588958	-1.598530

Total Energy -471.7067281 Ha

Vibrational frequencies of H_2O, intensities

Mode	au_amu	cm-1	km/mol
16	0.330782	1700.4	203.51
17	0.675082	3470.3	461.97
18	0.733832	3772.3	14.27

29.1 Na_2Pt^-

Final Coordinates (Angstroms)

	ATOM	X	Y	Z
1	Pt	-0.163561	-0.326704	-0.299786
2	Na	2.407004	-1.230003	0.084821
3	Na	1.214782	1.235747	1.493303

Total Energy -519.9642025 Ha

29.2 $Na_2PtH_2O^-$

Reaction precursor

Final Coordinates (Angstroms)

	ATOM	X	Y	Z
1	Pt	-0.187710	-0.306546	-0.294350
2	Na	2.454425	-1.275238	0.069766
3	Na	1.191510	1.260824	1.502923
4	H	0.287837	1.625903	-0.563911
5	H	1.472430	2.549611	-1.079378
6	O	0.714638	2.638416	-0.472880

Total Energy -596.4844693 Ha

Vibrational frequencies of H_2O, intensities

Mode	au_amu	cm-1	km/mol
16	0.285023	1465.2	363.44
17	0.319795	1643.9	756.50
18	0.721538	3709.1	1.03

Intermediate state

$coordinates

Pt	-0.08268611177313	-0.42525558245374	-1.12596029239285
Na	2.95621898689671	-3.59897215551067	2.45353488113589
Na	0.81261215189125	2.19952278035628	3.63307892472077
H	1.00698048994668	2.31875567571210	-0.60647257185324
H	4.00433273344582	4.60227551620785	0.62819687850398
O	2.41201280393460	5.14059712036261	1.38044580358183

Total Energy -596.463854 Ha

30.1 $NaPt_2^-$

Final Coordinates (Angstroms)

ATOM		X	Y	Z
1	Pt	-0.477564	0.108659	0.128437
2	Na	2.105521	0.434465	-0.457643
3	Pt	1.128972	0.860956	1.993621

Total Energy -553.1005673 Ha

30.2 $NaPt_2H_2O^-$

Reaction precursor

Final Coordinates (Angstroms)

ATOM		X	Y	Z
1	Pt	-0.493112	0.107284	0.157692
2	Na	2.147675	0.429610	-0.477374
3	Pt	1.103452	0.846069	2.015992
4	H	0.437934	2.176848	-0.775548
5	H	1.365925	2.653891	0.284470
6	O	1.246149	2.771216	-0.702560

Total Energy -629.6122206 Ha

Vibrational frequencies of H_2O, intensities

Mode	au_amu	cm-1	km/mol
16	0.317003	1629.6	213.06
17	0.605890	3114.6	180.80
18	0.624129	3208.3	424.98

Intermediate state

$coordinates

Pt	-0.89513919218836	0.74987905355958	0.32148054853461
Na	3.86357275837548	3.10368353451399	-0.18403763240819
Pt	2.06182156134529	0.93762284707972	3.95092187896615
H	0.54851002287342	1.76748234412063	-2.24638788054174
H	1.17247295298681	4.54320850923537	-4.74608163152592
O	2.57907136238302	3.51208822502074	-3.98599822129755

Total Energy -629.592754 Ha

31.1 P_2Pt^-

Final Coordinates (Angstroms)

	ATOM	X	Y	Z
1	P	-0.868659	0.190162	0.349825
2	Pt	2.968904	0.070065	0.128892
3	P	1.016356	0.494270	0.909267

Total Energy -878.1419187 Ha

31.2 $P_2PtH_2O^-$

Final Coordinates (Angstroms)

	ATOM	X	Y	Z
1	Pt	-0.827130	0.224092	0.550856
2	P	3.001091	-0.119891	0.693514
3	P	1.100624	-0.579735	1.058474
4	H	0.175164	2.070573	-0.839583
5	H	1.656466	2.234826	-1.010028
6	O	0.826914	2.663106	-1.291062

Total Energy -954.6219904 Ha

Vibrational frequencies of H_2O, intensities

Mode	au_amu	cm-1	km/mol
16	0.323540	1663.2	164.47
17	0.662660	3406.4	453.65
18	0.725281	3728.3	2.92

32.1 Ni_3^-

Final Coordinates (Angstroms)

	ATOM	X	Y	Z
1	Ni	-0.131759	-0.146655	0.044253
2	Ni	2.143471	-0.201323	-0.053498
3	Ni	1.103777	0.535268	1.834356

Total Energy -580.2077339 Ha

32.2 $Ni_3H_2O^-$

In this method, the structural optimization of $Ni_3H_2O^-$ did not be completed successfully, because SCF iterations are always not converging.

33.1 Ni_2Pt^-

Final Coordinates (Angstroms)

	ATOM	X	Y	Z
1	Ni	-0.138846	-0.016202	-0.029805

2	Pt	2.285128	-0.137640	-0.253205
3	Ni	0.970319	0.908338	1.670995

Total Energy -582.5250013 Ha

33.2 $Ni_2PtH_2O^-$

In this method, the structural optimization of $Ni_2PtH_2O^-$ did not be completed successfully too, because SCF iterations are always not converging.

34.1 Cu_3 $^-$

Final Coordinates (Angstroms)

ATOM		X	Y	Z
1	Cu	-0.225678	-0.069845	-0.128488
2	Cu	2.253010	-0.111945	-0.205936
3	Cu	1.089269	0.936286	1.722408

Total Energy -667.7009676 Ha

34.2 $Cu_3H_2O^-$

Final Coordinates (Angstroms)

ATOM		X	Y	Z
1	Cu	-0.236121	-0.158224	-0.004258
2	Cu	2.242687	-0.217366	-0.112152
3	Cu	1.109080	0.569929	1.945264
4	H	0.164578	2.680920	-0.437335
5	H	1.644597	2.781496	-0.306908
6	O	0.883202	3.328161	-0.581938

Total Energy -744.1779319 Ha

Vibrational frequencies of H_2O, intensities

Mode	au_amu	cm-1	km/mol
16	0.323624	1663.6	273.41
17	0.709644	3647.9	140.14
18	0.721326	3708.0	7.25

35.1 Cu_2Pt $^-$

Final Coordinates (Angstroms)

ATOM		X	Y	Z
1	Cu	-0.214178	-0.051076	-0.093960
2	Pt	2.278016	-0.117011	-0.215256
3	Cu	1.052762	0.922584	1.697201

Total Energy -640.5435557 Ha

35.2 $Cu_2PtH_2O^-$

Final Coordinates (Angstroms)

ATOM		X	Y	Z
1	Cu	-0.116373	-0.817762	0.187820
2	Pt	2.293274	-0.174454	0.117829
3	Cu	0.854214	0.148513	2.143650
4	H	0.471430	2.768057	-0.378836
5	H	1.698547	1.944355	-0.704253
6	O	1.274144	2.831746	-0.924232

Total Energy -717.0276033 Ha

Vibrational frequencies of H_2O, intensities

Mode	au_amu	cm-1	km/mol
16	0.320820	1649.2	157.88
17	0.596138	3064.4	1059.06
18	0.730821	3756.8	7.74

36.1 As_2Pt^-

Final Coordinates (Angstroms)

ATOM		X	Y	Z
1	As	-0.474432	-0.000634	-0.001167
2	Pt	2.521380	-0.058569	-0.107744
3	As	1.069652	0.813700	1.496895

Total Energy -849.9043740 Ha

36.2 $As_2PtH_2O^-$

Final Coordinates (Angstroms)

ATOM		X	Y	Z
1	Pt	-0.517657	-0.107983	0.205252
2	As	2.662334	-0.694951	0.280919
3	As	1.159124	0.160382	1.792941
4	H	0.203002	2.031936	-0.843717
5	H	1.650491	2.371895	-1.020540
6	O	0.775835	2.731691	-1.252685

Total Energy -926.3852044 Ha

Vibrational frequencies of H_2O, intensities

Mode	au_amu	cm-1	km/mol
16	0.323541	1663.2	193.71
17	0.652235	3352.8	561.55
18	0.726865	3736.4	4.33

37.1 Sr_2Cd^-

Final Coordinates (Angstroms)

ATOM	X	Y	Z

1	Cd	-0.582136	-0.675688	-0.184290
2	Cd	2.607619	-0.715242	-0.345553
3	Sr	1.095783	1.170325	1.727427

Total Energy -230.5499766 Ha

37.2 $Sr_2CdH_2O^-$

Final Coordinates (Angstroms)

ATOM		X	Y	Z
1	Cd	-0.677689	-0.758483	-0.289305
2	Sr	2.790679	-1.158712	-0.790604
3	Sr	1.116285	1.435592	2.064118
4	H	0.151003	2.084624	-0.652492
5	H	1.660138	2.179512	-0.769520
6	O	0.892714	2.710437	-0.400026

Total Energy -269.1136980 Ha

Vibrational frequencies of H_2O, intensities

Mode	au_amu	cm-1	km/mol
16	0.297177	1527.6	0.49
17	0.590201	3033.9	285.55
18	0.610469	3138.1	1224.82

38.1 Sr_2Re^-

Final Coordinates (Angstroms)

ATOM		X	Y	Z
1	Re	-0.507112	-0.315738	-0.189564
2	Sr	2.773311	-0.547942	-0.772399
3	Sr	0.930976	1.471296	2.235864

Total Energy -254.0478953 Ha

38.2 $Sr_2ReH_2O^-$

Reaction precursor

Final Coordinates (Angstroms)

ATOM		X	Y	Z
1	Re	-0.399035	-0.252028	-0.107480
2	Sr	2.689071	-0.507224	-0.708102
3	Sr	0.907139	1.366868	2.089483
4	H	0.329271	2.186603	-0.331816
5	H	1.597538	3.074760	-0.397765
6	O	0.684039	3.115938	-0.041648

Total Energy -330.5876449 Ha

Vibrational frequencies of H_2O, intensities

Mode	au_amu	cm-1	km/mol
16	0.299599	1540.1	60.36
17	0.487389	2505.4	694.71
18	0.691274	3553.5	128.21

Intermediate state
$coordinates

Re	-0.96544230660963	-0.22858404052800	-0.38864524360201
Sr	5.05522499533912	0.05948032031163	-1.49777750784426
Sr	1.79383949955995	1.92443594927388	4.84251195195271
H	0.31022926469531	1.75705119575040	-2.26032010938594
H	2.20373763684383	4.99613050607961	-1.85232676690317
O	2.38586177226209	3.87459446127758	-3.33738773128588

Total Energy -330.532855 Ha

39.1 Sr_2Pt^-

Final Coordinates (Angstroms)

	ATOM	X	Y	Z
1	Sr	-0.556958	-0.296179	-0.544856
2	Pt	2.273760	-0.119924	-0.220615
3	Sr	1.399798	1.170600	2.153456

Total Energy -298.1838754 Ha

39.2 $Sr_2PtH_2O^-$
Reaction precursor

Final Coordinates (Angstroms)

	ATOM	X	Y	Z
1	Pt	-0.312302	-0.257826	-0.144195
2	Sr	2.378716	-1.316678	-0.682251
3	Sr	0.989506	1.242194	1.892178
4	H	0.340870	1.717760	-0.581328
5	H	1.692294	2.464920	-0.886745
6	O	0.844045	2.642601	-0.435489

Total Energy -374.7290421 Ha

Vibrational frequencies of H_2O, intensities

Mode	au_amu	cm-1	km/mol
16	0.301994	1552.4	66.30
17	0.418145	2149.5	573.20
18	0.710526	3652.5	38.67

Intermediate state
$coordinates

Pt	-0.66730031224542	-0.15992463067724	-0.68239173877894
Sr	4.41944897903755	-2.86946999419046	-0.95355634313344

Sr	1.81460673362769	2.02080499722079	3.84297200162044
H	0.36209410021706	2.59563049951357	-1.04650631149213
H	4.36537357024068	4.43994656241534	-0.50287220909930
O	3.19695778176546	4.93465352760047	0.79558923628538

Total Energy -374.685279 Ha

40.1 $SrCd_2^-$

Final Coordinates (Angstroms)

	ATOM	X	Y	Z
1	Cd	-0.582136	-0.675688	-0.184290
2	Cd	2.607619	-0.715242	-0.345553
3	Sr	1.095783	1.170325	1.727427

Total Energy -230.5499766 Ha

40.2 $SrCd_2H_2O^-$

Final Coordinates (Angstroms)

	ATOM	X	Y	Z
1	Cd	-0.607488	-0.845552	-0.365012
2	Cd	2.599145	-0.852779	-0.492331
3	Sr	1.129609	1.477726	2.054928
4	H	0.163695	2.111423	-0.728354
5	H	1.666360	1.963161	-0.801085
6	O	0.981808	2.638992	-0.505975

Total Energy -307.6089677 Ha

Vibrational frequencies of H_2O, intensities

Mode	au_amu	cm-1	km/mol
16	0.298930	1536.6	7.37
17	0.583544	2999.7	583.69
18	0.616796	3170.6	1229.28

41.1 $SrRe_2^-$

Final Coordinates (Angstroms)

	ATOM	X	Y	Z
1	Sr	-0.395350	0.382133	-0.407893
2	Re	2.036407	-0.173826	0.495441
3	Re	0.705472	0.886638	1.953442

Total Energy -353.6116957 Ha

41.2 $SrRe_2H_2O^-$

Final Coordinates (Angstroms)

ATOM	X	Y	Z

1	Sr	-0.639796	0.380031	-0.648422
2	Re	2.139002	-0.160568	0.628344
3	Re	0.847323	0.875483	2.061068
4	H	0.704913	2.697756	0.147561
5	H	1.547596	2.304759	-1.018987
6	O	0.744012	2.851032	-0.847094

Total Energy -430.6243457 Ha

Vibrational frequencies of H_2O, intensities

Mode	au_amu	cm-1	km/mol
16	0.313018	1609.1	124.63
17	0.594885	3058.0	368.00
18	0.675521	3472.5	97.75

42.1 $SrPt_2^-$

Final Coordinates (Angstroms)

	ATOM	X	Y	Z
1	Pt	-0.678227	0.181671	-0.114613
2	Sr	1.964160	0.194405	-0.380295
3	Pt	1.146681	0.920848	2.034875

Total Energy -442.2345332 Ha

42.2 $SrPt_2H_2O^-$

Reaction precursor

Final Coordinates (Angstroms)

	ATOM	X	Y	Z
1	Pt	-0.670008	0.176969	-0.141033
2	Sr	2.030565	0.196331	-0.360413
3	Pt	1.064211	0.933294	2.050388
4	H	0.385689	2.208854	-0.811755
5	H	1.291709	2.676759	0.294831
6	O	1.240884	2.756285	-0.709547

Total Energy -518.7367380 Ha

Vibrational frequencies of H_2O, intensities

Mode	au_amu	cm-1	km/mol
16	0.312367	1605.7	166.43
17	0.555747	2856.8	301.81
18	0.598802	3078.1	450.96

Intermediate state

$coordinates

Pt	-1.30842019623873	0.47298254893028	-0.12181298945688
Sr	3.92671495217760	0.57742689738672	-0.77398731018823
Pt	2.12108410583882	1.58911621067110	3.93272748623059

H	-0.57855084034545	2.48840509970049	-2.17205593049322
H	1.44311650126714	5.17364690905922	-1.28079250233653
O	1.83149161418876	4.38996481063126	-2.91694265455318

Total Energy -0.6779664 Ha

43.1 Tc_2Pt^-

Final Coordinates (Angstroms)

	ATOM	X	Y	Z
1	Tc	-1.193587	0.324336	0.596654
2	Pt	3.331738	0.108503	0.199604
3	Tc	0.978450	0.321657	0.591726

Total Energy -415.3142247 Ha

43.2 $Tc_2PtH_2O^-$

Final Coordinates (Angstroms)

	ATOM	X	Y	Z
1	Tc	-1.195088	-0.028050	0.469521
2	Pt	3.176122	-0.548569	0.166074
3	Tc	0.887415	-0.377082	0.901011
4	H	1.632849	2.783119	0.107845
5	H	2.933383	2.020771	0.137367
6	O	2.592437	2.949557	0.107640

Total Energy -491.7924919 Ha

Vibrational frequencies of H_2O, intensities

Mode	au_amu	cm-1	km/mol
16	0.322327	1656.9	198.62
17	0.658918	3387.2	471.12
18	0.727629	3740.4	6.29

44.1 Pd_2Pt^-

Final Coordinates (Angstroms)

	ATOM	X	Y	Z
1	Pd	-0.309321	-0.104124	-0.191548
2	Pt	2.307349	-0.128002	-0.235475
3	Pd	1.118573	0.986623	1.815008

Total Energy -522.2252998 Ha

44.2 $Pd_2PtH_2O^-$

Final Coordinates (Angstroms)

	ATOM	X	Y	Z
1	Pd	-0.270018	-0.619721	0.084749

2	Pt	2.312077	-0.211099	0.054913
3	Pd	1.026532	0.193188	2.294331
4	O	1.229043	2.833246	-0.886326
5	H	0.380874	2.609899	-0.461655
6	H	1.727375	1.993056	-0.664813

Total Energy -598.7099459 Ha

Vibrational frequencies of H_2O, intensities

Mode	au_amu	cm-1	km/mol
16	0.323229	1661.6	199.72
17	0.615301	3163.0	711.04
18	0.724705	3725.3	4.01

45.1 Cd_3^-

Final Coordinates (Angstroms)

ATOM		X	Y	Z
1	Cd	-0.593222	-0.280551	-0.161553
2	Cd	2.559951	-0.293138	-0.301596
3	Cd	1.135036	0.647885	2.370764

Total Energy -269.1587289 Ha

45.2 $Cd_3H_2O^-$

Final Coordinates (Angstroms)

ATOM		X	Y	Z
1	Cd	-0.683929	-0.294189	-0.196579
2	Cd	2.655056	-0.313078	-0.360383
3	Cd	1.130637	0.681464	2.464577
4	H	0.171971	2.716146	-0.470121
5	H	1.645933	2.815716	-0.325446
6	O	0.888354	3.378859	-0.609376

Total Energy -346.1064320 Ha

Vibrational frequencies of H_2O, intensities

Mode	au_amu	cm-1	km/mol
16	0.316777	1628.4	129.73
17	0.676491	3477.5	510.19
18	0.677364	3482.0	709.46

46.1 Cd_2Re^-

Final Coordinates (Angstroms)

ATOM		X	Y	Z
1	Cd	-0.542561	-0.242130	-0.445426
2	Re	2.383697	-0.149918	-0.275791
3	Cd	1.275465	1.146544	2.109202

Total Energy -331.0638982 Ha

46.2 $Cd_2ReH_2O^-$

Final Coordinates (Angstroms)

ATOM		X	Y	Z
1	Re	-0.582469	-0.677207	0.061273
2	Cd	2.493435	-0.812151	-0.209227
3	Cd	1.034016	0.669870	2.347841
4	H	0.363327	1.995586	-0.875584
5	H	1.767833	2.528451	-0.964951
6	O	0.856988	2.788422	-1.197182

Total Energy -407.5727121 Ha

Vibrational frequencies of H_2O, intensities

Mode	au_amu	cm-1	km/mol
16	0.319682	1643.3	170.80
17	0.667780	3432.7	720.60
18	0.721044	3706.5	2.82

47.1 Cd_2Pt^-

Final Coordinates (Angstroms)

ATOM		X	Y	Z
1	Cd	-0.618855	-0.493817	-0.908435
2	Pt	1.896897	-0.021206	-0.039010
3	Cd	1.838558	1.269520	2.335430

Total Energy -375.1870749 Ha

47.2 $Cd_2PtH_2O^-$

Final Coordinates (Angstroms)

ATOM		X	Y	Z
1	Cd	-0.530789	-0.855867	-0.671761
2	Pt	1.936780	-0.031924	0.126141
3	Cd	1.856627	0.861062	2.685233
4	O	1.367793	2.963097	-1.191028
5	H	1.642181	2.127604	-0.694662
6	H	0.565899	2.640529	-1.636370

Total Energy -451.6694855 Ha

Vibrational frequencies of H_2O, intensities

Mode	au_amu	cm-1	km/mol
16	0.316926	1629.2	179.46
17	0.587707	3021.1	1103.08
18	0.731792	3761.8	7.52

48.1 $CdRe_2^-$

Final Coordinates (Angstroms)

	ATOM	X	Y	Z
1	Cd	-0.653416	-0.170152	-0.313014
2	Re	2.407511	-0.002810	-0.005169
3	Re	1.362506	0.927458	1.706167

Total Energy -392.6115003 Ha

48.2 $CdRe_2H_2O^-$

Final Coordinates (Angstroms)

	ATOM	X	Y	Z
1	Cd	-0.854236	-0.955857	0.182299
2	Re	2.285159	-0.533564	0.184653
3	Re	1.486958	0.458002	1.929702
4	H	0.239999	2.459927	-1.170982
5	H	1.655951	2.192992	-0.747759
6	O	1.119299	2.871472	-1.215743

Total Energy -469.1013324 Ha

Vibrational frequencies of H_2O, intensities

Mode	au_amu	cm-1	km/mol
16	0.321213	1651.2	211.02
17	0.687807	3535.7	328.19
18	0.733638	3771.3	14.01

49.1 Sb_2Pt^-

Final Coordinates (Angstroms)

	ATOM	X	Y	Z
1	Sb	-0.349817	-0.155876	-0.286753
2	Pt	2.257267	-0.114590	-0.210803
3	Sb	1.209151	1.024963	1.885540

Total Energy -477.3295495 Ha

49.2 $Sb_2PtH_2O^-$

Final Coordinates (Angstroms)

	ATOM	X	Y	Z
1	Pt	-0.232245	-0.293156	0.057244
2	Sb	2.331203	-0.895016	-0.060353
3	Sb	1.082511	0.286453	2.262860
4	H	0.314851	1.974612	-0.961407
5	H	1.653189	2.631435	-0.863759
6	O	0.783620	2.788642	-1.272415

Total Energy -553.8087061 Ha

Vibrational frequencies of H_2O, intensities

Mode	au_amu	cm-1	km/mol
16	0.323166	1661.2	196.54
17	0.663331	3409.8	498.88
18	0.728800	3746.4	0.97

50.1 $Re_3{}^-$

Final Coordinates (Angstroms)

	ATOM	X	Y	Z
1	Re	-0.339069	-0.239783	0.283453
2	Re	2.478469	-0.231078	0.253168
3	Re	1.046311	0.518944	1.843926

Total Energy -453.6481074 Ha

50.2 $Re_3H_2O^-$

Final Coordinates (Angstroms)

	ATOM	X	Y	Z
1	Re	-0.205949	-0.255828	0.247660
2	Re	2.364518	-0.256969	0.201065
3	Re	1.027142	0.560880	1.931823
4	H	0.167780	2.663989	-0.613932
5	H	1.626669	2.905977	-0.470874
6	O	0.827862	3.366868	-0.793070

Total Energy -530.6017609 Ha

Vibrational frequencies of H_2O, intensities

Mode	au_amu	cm-1	km/mol
16	0.324959	1670.4	270.93
17	0.700235	3599.5	169.86
18	0.717713	3689.4	6.84

51.1 Re_2Pt^-

Final Coordinates (Angstroms)

	ATOM	X	Y	Z
1	Re	-0.263456	0.012083	0.022228
2	Pt	2.433787	-0.130695	-0.240430
3	Re	0.946269	0.873109	1.606186

Total Energy -498.1585425 Ha

51.2 $Re_2PtH_2O^-$

Final Coordinates (Angstroms)

ATOM		X	Y	Z
1	Pt	-0.986421	0.063306	0.320856
2	Re	3.102258	-0.562813	0.515660
3	Re	1.150065	-0.039262	1.424340
4	H	0.172291	2.066959	-0.878298
5	H	1.660220	2.268607	-0.964735
6	O	0.834716	2.696174	-1.255654

Total Energy -574.6705625 Ha

Vibrational frequencies of H_2O, intensities

Mode	au_amu	cm-1	km/mol
16	0.322905	1659.9	171.72
17	0.664199	3414.3	368.55
18	0.726555	3734.8	15.97

52.1 Ir_2Pt^-

Final Coordinates (Angstroms)

ATOM		X	Y	Z
1	Ir	-0.242578	-0.054233	-0.099769
2	Pt	2.303767	-0.124151	-0.228390
3	Ir	1.055412	0.932881	1.716144

Total Energy -551.2979253 Ha

52.2 $Ir_2PtH_2O^-$

Final Coordinates (Angstroms)

ATOM		X	Y	Z
1	Pt	-0.304898	-0.384846	0.173480
2	Ir	2.234624	-0.359942	-0.007983
3	Ir	1.203460	0.288729	2.111307
4	H	0.198192	2.123728	-0.918783
5	H	1.675697	2.139671	-0.934998
6	O	0.926055	2.685630	-1.260852

Total Energy -627.7802424 Ha

Vibrational frequencies of H_2O, intensities

Mode	au_amu	cm-1	km/mol
16	0.323185	1661.3	311.65
17	0.690118	3547.5	239.96
18	0.699228	3594.4	28.63

53.1 Pt_3^-

Final Coordinates (Angstroms)

ATOM		X	Y	Z

1	Pt	-0.267679	-0.081184	-0.149347
2	Pt	2.292157	-0.122073	-0.224568
3	Pt	1.092123	0.957754	1.761900

Total Energy -585.8107647 Ha

53.2 $Pt_3H_2O^-$

Final Coordinates (Angstroms)

	ATOM	X	Y	Z
1	Pt	-0.243461	0.327582	-0.099063
2	Pt	2.361605	0.301848	-0.088850
3	Pt	1.049239	0.390423	2.145518
4	H	0.357175	2.829309	-0.559636
5	H	1.832766	2.830854	-0.585342
6	O	1.091225	3.446598	-0.788496

Total Energy -662.6860640 Ha

Vibrational frequencies of H_2O, intensities

Mode	au_amu	cm-1	km/mol
16	0.322992	1660.3	296.11
17	0.678677	3488.7	238.87
18	0.683137	3511.7	69.16

54.1 $PtAu_2{}^-$

Final Coordinates (Angstroms)

	ATOM	X	Y	Z
1	Pt	-0.008131	0.691099	0.987800
2	Au	-0.367220	1.630662	-1.360095
3	Au	0.347588	-0.246677	3.336897

Total Energy -618.5740399 Ha

54.2 $PtAu_2H_2O^-$

Final Coordinates (Angstroms)

	ATOM	X	Y	Z
1	Pt	0.803855	0.024047	0.650938
2	Au	2.074868	-0.245245	-1.624779
3	Au	-0.459211	0.520964	2.883103
4	H	0.623938	2.863605	0.162327
5	H	1.330269	2.497707	-1.100927
6	O	0.969329	3.287414	-0.648192

Total Energy -695.4278365 Ha

Vibrational frequencies of H_2O, intensities

Mode	au_amu	cm-1	km/mol
16	0.321528	1652.8	253.16

17	0.700977	3603.4	136.63
18	0.715216	3676.6	16.75

55.1 Be_3Pt^-

Final Coordinates (Angstroms)

	ATOM	X	Y	Z
1	Pt	2.716752	-0.025996	0.688186
2	Be	1.560623	-0.320361	-1.043849
3	Be	1.932397	1.651411	-0.429299
4	Be	0.772160	0.695278	1.018350

Total Energy -239.3169752 Ha

55.2 $Be_3PtH_2O^-$

Final Coordinates (Angstroms)

	ATOM	X	Y	Z
1	Pt	2.733062	-0.005777	0.672816
2	Be	1.529247	-0.366039	-1.034050
3	Be	1.967942	1.694170	-0.401833
4	Be	0.751680	0.677978	0.996454
5	H	2.700209	2.668157	2.351337
6	H	3.028224	2.047147	3.675966
7	O	3.024259	2.920099	3.245798

Total Energy -316.1487893 Ha

Vibrational frequencies of H_2O, intensities

Mode	au_amu	cm-1	km/mol
19	0.321114	1650.7	247.74
20	0.687167	3532.4	421.75
21	0.730183	3753.5	10.86

56.1 C_3Pt^-

Final Coordinates (Angstroms)

	ATOM	X	Y	Z
1	Pt	2.777313	-0.242002	0.513888
2	C	1.305553	0.173217	-0.795526
3	C	2.070707	1.571187	1.046278
4	C	1.227598	1.340747	-0.037854

Total Energy -309.2803062 Ha

56.2 $C_3PtH_2O^-$

Final Coordinates (Angstroms)

	ATOM	X	Y	Z
1	Pt	2.779896	-0.250375	0.511726

2	C	1.292851	0.175856	-0.807125
3	C	2.058048	1.594187	1.049444
4	C	1.250377	1.323481	-0.027259
5	H	2.950241	2.129734	4.094079
6	H	2.639069	2.589521	2.679463
7	O	2.897754	2.950195	3.576093

Total Energy -386.0872380 Ha

Vibrational frequencies of H_2O, intensities

Mode	au_amu	cm-1	km/mol
19	0.329357	1693.1	117.84
20	0.623933	3207.3	1386.96
21	0.733355	3769.8	20.12

57.1 N_3Pt^-

Final Coordinates (Angstroms)

ATOM		X	Y	Z
1	N	1.633808	0.257871	2.751214
2	N	1.215365	-0.004458	-0.981372
3	Pt	1.401671	0.136314	0.833392
4	N	1.780688	0.318898	3.893310

Total Energy -359.4084444 Ha

57.2 $N_3PtH_2O^-$

Final Coordinates (Angstroms)

ATOM		X	Y	Z
1	N	1.685990	-0.528314	2.300526
2	N	1.818640	-0.720487	1.030105
3	Pt	0.974832	1.096629	0.589899
4	N	1.117356	0.623452	2.581293
5	H	2.341612	2.065646	3.179896
6	H	3.013068	2.987116	2.211964
7	O	2.939475	2.857503	3.173892

Total Energy -436.1608089 Ha

Vibrational frequencies of H_2O, intensities

Mode	au_amu	cm-1	km/mol
19	0.328373	1688.0	238.63
20	0.658662	3385.8	561.72
21	0.727854	3741.5	4.84

58.1 F_3Pt^-

Final Coordinates (Angstroms)

ATOM	X	Y	Z

1	Pt	1.656040	0.989410	0.184394
2	F	2.627984	0.175719	-1.368016
3	F	3.257469	1.828883	0.941747
4	F	0.360381	1.635928	1.572991

Total Energy -494.7405063 Ha

58.2 $F_3PtH_2O^-$

Final Coordinates (Angstroms)

ATOM		X	Y	Z
1	Pt	1.638593	0.551578	0.371990
2	F	2.154587	-0.340223	-1.353261
3	F	3.163351	1.764069	0.680945
4	F	0.752934	1.193839	2.089611
5	H	2.020561	1.936575	3.325639
6	H	3.341511	2.290292	2.762481
7	O	2.875123	2.324822	3.619534

Total Energy -571.5872546 Ha

Vibrational frequencies of H_2O, intensities

Mode	au_amu	cm-1	km/mol
19	0.332309	1708.2	254.72
20	0.693559	3565.2	289.90
21	0.720989	3706.2	57.84

59.1 Na_4^-

Final Coordinates (Angstroms)

ATOM		X	Y	Z
1	Na	3.325488	0.047944	3.873781
2	Na	-1.494527	0.461962	1.429930
3	Na	1.634771	1.016229	1.356719
4	Na	0.130316	-0.152768	4.153591

Total Energy -648.4492325 Ha

59.2 $Na_4H_2O^-$

Final Coordinates (Angstroms)

ATOM		X	Y	Z
1	Na	3.696394	-0.414510	3.843492
2	Na	0.568211	-0.345284	-2.012684
3	Na	1.486914	0.234647	1.043702
4	Na	0.214362	0.597092	4.515585
5	H	1.917765	2.811907	2.849128
6	H	3.315624	2.379502	2.501646
7	O	2.691703	3.118191	2.326707

Total Energy -725.5986618 Ha

Vibrational frequencies of H_2O, intensities

Mode	au_amu	cm-1	km/mol
19	0.315095	1619.7	207.50
20	0.684077	3516.5	403.03
21	0.691653	3555.4	13.77

Na_3Pt^- (Catalyst)

Final Coordinates (Angstroms)

	ATOM	X	Y	Z
1	Pt	3.310450	0.204546	1.373910
2	Na	1.407775	-0.344687	-0.732644
3	Na	3.201930	2.147441	-0.766009
4	Na	0.975526	1.896195	1.360449

Total Energy -682.2641035 Ha

60.1 Na_3Pt^-

Final Coordinates (Angstroms)

	ATOM	X	Y	Z
1	Pt	3.310450	0.204546	1.373910
2	Na	1.407775	-0.344687	-0.732644
3	Na	3.201930	2.147441	-0.766009
4	Na	0.975526	1.896195	1.360449

Total Energy -682.2641035 Ha

60.2 $Na_3PtH_2O^-$

Reaction precursor

Final Coordinates (Angstroms)

	ATOM	X	Y	Z
1	Pt	3.311679	0.224142	1.368580
2	Na	1.388190	-0.371123	-0.794494
3	Na	3.256803	2.138187	-0.829244
4	Na	0.929036	1.891784	1.397336
5	H	2.476327	1.261471	2.996187
6	H	2.631573	2.633546	3.731494
7	O	1.953054	1.942945	3.627081

Total Energy -758.7899947 Ha

Vibrational frequencies of H_2O, intensities

Mode	au_amu	cm-1	km/mol
19	0.302967	1557.4	52.93
20	0.403908	2076.3	1145.39
21	0.724370	3723.6	5.58

Intermediate state
$coordinates

Pt	6.33312792312711	0.44645457925680	2.71382781550426
Na	2.10714872304172	-0.72385597713272	-1.82232818858948
Na	6.02178454204892	3.34016895082814	-1.91640339247958
Na	1.82105697171493	3.41715056146556	2.70470741518939
H	5.20245165478317	2.27013412422635	4.80312163321905
H	5.02942043550300	5.75265505367829	5.97071472375658
O	3.57852413397996	4.61640958858918	6.29175835438301

Total Energy -758.758054 Ha

61.1 $Na_2Pt_2^-$

Final Coordinates (Angstroms)

	ATOM	X	Y	Z
1	Na	3.159130	-1.025049	2.111225
2	Pt	0.699389	-1.070390	1.006439
3	Pt	2.569904	1.165183	0.649527
4	Na	0.485931	1.250022	2.362492

Total Energy -715.4160394 Ha

61.2 $Na_2Pt_2H_2O^-$
Reaction precursor

Final Coordinates (Angstroms)

	ATOM	X	Y	Z
1	Na	3.199198	-1.061231	2.116859
2	Pt	0.742076	-0.999906	0.989351
3	Pt	2.583419	1.109604	0.611668
4	Na	0.389661	1.271299	2.411806
5	H	2.663700	2.681760	3.703072
6	H	2.335742	2.431233	2.169536
7	O	1.977177	2.948786	3.065285

Total Energy -791.9300242 Ha

Vibrational frequencies of H_2O, intensities

Mode	au_amu	cm-1	km/mol
19	0.295163	1517.3	116.90
20	0.332125	1707.3	1717.37
21	0.719695	3699.6	7.75

Intermediate state
$coordinates

Na	6.10532700022312	-2.21514252896092	3.85458121470563
Pt	1.38804211898991	-1.86465617918103	1.88230608512818
Pt	4.97147844107424	2.05799034571728	1.16997005797741
Na	0.73867978065241	2.62857575989930	4.68660868303590

H	4.49312944033530	5.02831434478697	7.15942309434010
H	4.68456771079077	4.02680821190042	3.41995160423488
O	3.04906589148226	5.60564826008943	6.14570086282332

Total Energy -791.916048 Ha

62.1 $NaPt_3^-$

Final Coordinates (Angstroms)

	ATOM	X	Y	Z
1	Pt	2.296180	-0.728541	2.814646
2	Pt	0.955204	-1.175557	0.492389
3	Pt	2.540033	0.875905	0.777626
4	Na	0.178277	0.777239	2.128465

Total Energy -748.0278940 Ha

62.2 $NaPt_3H_2O^-$

Final Coordinates (Angstroms)

	ATOM	X	Y	Z
1	Pt	2.360405	-0.934700	2.552945
2	Na	0.635001	-1.148522	0.388509
3	Pt	2.521265	0.920062	0.816337
4	Pt	0.243940	0.818552	2.255204
5	H	2.123543	3.477297	0.465031
6	H	0.982619	2.527823	0.163054
7	O	1.638603	3.121046	-0.305737

Total Energy -825.0209962 Ha

Vibrational frequencies of H_2O, intensities

Mode	au_amu	cm-1	km/mol
19	0.312661	1607.2	175.79
20	0.626193	3218.9	331.75
21	0.712179	3661.0	11.60

63.1 P_3Pt^-

Final Coordinates (Angstroms)

	ATOM	X	Y	Z
1	Pt	2.013657	-0.478884	1.761629
2	P	2.574018	0.438934	-0.319209
3	P	1.956256	2.501366	0.108793
4	P	0.596468	0.821151	0.389707

Total Energy -1218.9345760 Ha

63.2 $P_3PtH_2O^-$

Final Coordinates (Angstroms)

ATOM		X	Y	Z
1	Pt	2.015795	-0.484882	1.769497
2	P	2.586084	0.437007	-0.327375
3	P	1.958677	2.507977	0.107836
4	P	0.579842	0.822464	0.390963
5	H	2.853786	1.378780	3.306237
6	H	2.869399	2.751135	2.701110
7	O	3.083078	2.308471	3.548672

Total Energy -1295.9951931 Ha

Vibrational frequencies of H_2O, intensities

Mode	au_amu	cm-1	km/mol
19	0.324458	1667.9	239.45
20	0.668283	3435.3	300.61
21	0.702219	3609.7	31.68

64.1 Ni_3Pt^-

Final Coordinates (Angstroms)

ATOM		X	Y	Z
1	Pt	2.845297	0.044345	1.595142
2	Ni	1.748425	0.251607	-0.642775
3	Ni	2.677138	2.160619	0.272562
4	Ni	0.689383	1.243049	1.150355

Total Energy -775.5741777 Ha

64.2 $Ni_3PtH_2O^-$

Final Coordinates (Angstroms)

ATOM		X	Y	Z
1	Pt	2.686892	-0.418687	1.280652
2	Ni	1.657091	0.126143	-1.027922
3	Ni	2.206375	1.878071	0.332928
4	Ni	0.318376	0.461239	0.948399
5	H	2.742783	1.665688	2.334367
6	H	3.873088	2.678124	2.083427
7	O	2.913784	2.682530	2.262384

Total Energy -852.5876373 Ha

Vibrational frequencies of H_2O, intensities

Mode	au_amu	cm-1	km/mol
19	0.302602	1555.5	100.36
20	0.513283	2638.5	638.42
21	0.719212	3697.1	12.43

65.1 $NiPt_3^-$

Final Coordinates (Angstroms)

ATOM		X	Y	Z
1	Pt	2.928528	0.130148	1.682284
2	Pt	1.811277	0.172289	-0.815723
3	Ni	2.667388	2.117208	0.346527
4	Pt	0.576468	1.365283	1.296721

Total Energy -779.2981354 Ha

65.2 $NiPt_3H_2O^-$

Final Coordinates (Angstroms)

ATOM		X	Y	Z
1	Pt	2.946427	-0.210329	1.225987
2	Pt	1.468901	-0.061979	-1.069510
3	Ni	2.784162	1.774798	-0.179600
4	Pt	0.739737	1.433244	1.097151
5	H	3.148333	1.663413	3.377506
6	H	2.039806	2.641229	3.236119
7	O	2.819296	2.480577	3.809287

Total Energy -856.2910999 Ha

Vibrational frequencies of H_2O, intensities

Mode	au_amu	cm-1	km/mol
19	0.320698	1648.5	339.83
20	0.694162	3568.3	311.49
21	0.702126	3609.3	6.12

66.1 Cu_4^-

Final Coordinates (Angstroms)

ATOM		X	Y	Z
1	Cu	-0.187847	-0.801282	0.323825
2	Cu	1.861143	0.131076	-0.618207
3	Cu	2.746128	2.178554	0.370183
4	Cu	0.697177	1.246149	1.312184

Total Energy -890.3080458 Ha

66.2 $Cu_4H_2O^-$

Final Coordinates (Angstroms)

ATOM		X	Y	Z
1	Cu	2.616064	-0.213070	1.075686
2	Cu	1.731800	-0.162596	-1.241395
3	Cu	2.592982	1.928507	-0.217427
4	Cu	0.453813	0.934734	0.592093
5	H	2.779879	1.628808	3.281373

6	H	2.699311	2.930140	2.557484
7	O	2.860774	2.589211	3.458674

Total Energy -966.7571603 Ha

Vibrational frequencies of H_2O, intensities

Mode	au_amu	cm-1	km/mol
19	0.321923	1654.8	354.01
20	0.700825	3602.6	253.41
21	0.716131	3681.3	33.43

67.1 Cu_3Pt^-

Final Coordinates (Angstroms)

	ATOM	X	Y	Z
1	Pt	3.023884	0.277269	0.641391
2	Cu	0.747758	-0.632744	0.283804
3	Cu	3.571219	2.552916	-0.181223
4	Cu	1.385665	1.566437	-0.526284

Total Energy -862.6442617 Ha

67.2 $Cu_3PtH_2O^-$

Final Coordinates (Angstroms)

	ATOM	X	Y	Z
1	Pt	3.128127	0.194816	0.709160
2	Cu	0.803097	-0.552931	0.273554
3	Cu	3.502212	2.490136	-0.169881
4	Cu	1.295089	1.631855	-0.595145
5	H	2.555794	1.252784	2.824021
6	H	2.124555	2.689253	2.945345
7	O	2.325750	1.929820	3.519435

Total Energy -939.6234751 Ha

Vibrational frequencies of H_2O, intensities

Mode	au_amu	cm-1	km/mol
19	0.322284	1656.7	192.89
20	0.632643	3252.1	676.34
21	0.729854	3751.8	7.52

68.1 As_3Pt^-

Final Coordinates (Angstroms)

	ATOM	X	Y	Z
1	Pt	1.637679	-1.494754	0.305406
2	As	1.482854	0.052644	-1.640814
3	As	2.640096	1.882391	-0.570970
4	As	0.743327	0.774203	0.519239

Total Energy -1175.0744106 Ha

68.2 $As_3PtH_2O^-$

Final Coordinates (Angstroms)

ATOM		X	Y	Z
1	Pt	2.776325	-0.107020	1.535333
2	As	1.293607	0.059816	-1.804704
3	As	2.736393	1.485539	-0.379408
4	As	0.620927	0.643073	0.486543
5	H	2.899911	1.742021	3.164207
6	H	2.529846	3.180965	2.890616
7	O	2.877615	2.631338	3.613900

Total Energy -1253.6503751 Ha

Vibrational frequencies of H_2O, intensities

Mode	au_amu	cm-1	km/mol
19	0.321250	1651.4	169.67
20	0.636838	3273.7	964.00
21	0.731766	3761.6	11.92

69.1 Sr_4^-

Final Coordinates (Angstroms)

ATOM		X	Y	Z
1	Sr	2.924547	-0.960743	2.577902
2	Sr	0.561456	-1.695076	0.344822
3	Sr	1.891139	1.485119	0.475892
4	Sr	-0.230202	0.367372	2.847320

Total Energy -204.5611702 Ha

69.2 $Sr_4H_2O^-$

Final Coordinates (Angstroms)

ATOM		X	Y	Z
1	Sr	3.198971	-0.963202	2.789479
2	Sr	0.369673	-1.849786	0.101517
3	Sr	2.014733	1.633890	0.299037
4	Sr	-0.436437	0.375769	3.055902
5	H	2.226281	2.858123	3.260434
6	H	3.617546	2.837647	2.669648
7	O	2.900206	3.489105	2.891559

Total Energy -281.9300822 Ha

Vibrational frequencies of H_2O, intensities

Mode	au_amu	cm-1	km/mol
19	0.302082	1552.9	127.22

20	0.635021	3264.3	93.18
21	0.640663	3293.3	1119.20

70.1 Sr_3Cd^-

Final Coordinates (Angstroms)

	ATOM	X	Y	Z
1	Cd	3.172673	-0.280595	1.229996
2	Sr	1.526481	-0.275588	-1.398003
3	Sr	3.327480	2.367527	-0.359820
4	Sr	0.566044	1.406997	1.430313

Total Energy -243.1784997 Ha

70.2 $Sr_3CdH_2O^-$

Final Coordinates (Angstroms)

	ATOM	X	Y	Z
1	Cd	3.262303	-0.335826	1.306380
2	Sr	1.413342	-0.588300	-1.733356
3	Sr	3.601965	2.655831	-0.407185
4	Sr	0.315068	1.486637	1.736648
5	H	2.638403	1.404148	3.653617
6	H	2.603367	2.858584	3.170515
7	O	2.112214	2.239879	3.770322

Total Energy -320.5283147 Ha

Vibrational frequencies of H_2O, intensities

Mode	au_amu	cm-1	km/mol
19	0.302493	1555.0	38.62
20	0.635316	3265.8	471.43
21	0.646828	3325.0	327.44

71.1 Sr_3Re^-

IS

Final Coordinates (Angstroms)

	ATOM	X	Y	Z
1	Sr	3.530163	-0.897655	1.803553
2	Sr	0.742894	-0.985468	-0.889845
3	Re	2.472086	1.379539	0.024041
4	Sr	0.294786	1.389658	2.216297

Total Energy -305.4461938 Ha

71.2 $Sr_3ReH_2O^-$

Reaction precursor

Final Coordinates (Angstroms)

ATOM		X	Y	Z
1	Sr	3.547203	-0.891340	1.807301
2	Sr	0.732278	-0.980525	-0.900977
3	Re	2.457049	1.362058	0.043680
4	Sr	0.303400	1.395883	2.204042
5	H	2.857701	2.142801	3.883194
6	H	2.659026	2.575643	2.424968
7	O	2.406838	2.845767	3.368885

Total Energy -382.0010238 Ha

Vibrational frequencies of H_2O, intensities

Mode	au_amu	cm-1	km/mol
19	0.301558	1550.2	150.33
20	0.566358	2911.4	572.83
21	0.696537	3580.5	127.34

Intermediate state

$coordinates

Sr	6.73367622088838	-0.42371685594893	3.93619128240811
Sr	2.76956618896030	-2.43980517235078	-1.53609350026832
Re	3.90021429849526	2.95008165155097	0.20512433034654
Sr	-0.29640513952382	1.72718011914809	3.92019600553819
H	9.01410081093386	5.37986758669389	4.01217673525710
H	6.47170320094215	3.25317125693577	1.30314509379095
O	9.42570415014554	3.77640903881494	3.22063486525335

Total Energy -381.935264 Ha

72.1 Sr_3Pt^-

Final Coordinates (Angstroms)

ATOM		X	Y	Z
1	Sr	2.963547	-0.226336	2.768458
2	Sr	0.600244	-1.503173	0.782721
3	Sr	2.077950	1.642670	0.199262
4	Pt	0.411520	0.834098	2.265307

Total Energy -348.8743125 Ha

72.2 $Sr_3PtH_2O^-$

Final Coordinates (Angstroms)

ATOM		X	Y	Z
1	Pt	2.925643	0.016276	1.013463
2	Sr	1.735121	-0.369271	-1.683363
3	Sr	2.993662	2.844072	0.398343
4	Sr	0.197323	0.622301	2.016584
5	H	3.205743	2.613921	3.469964

6	H	3.817985	1.523921	2.568208
7	O	4.004439	2.468467	2.919486

Total Energy -426.1548655 Ha

Vibrational frequencies of H_2O, intensities

Mode	au_amu	cm-1	km/mol
19	0.306106	1573.5	135.58
20	0.531607	2732.7	769.22
21	0.695044	3572.9	145.44

73.1 $Sr_2Re_2^-$

Final Coordinates (Angstroms)

ATOM		X	Y	Z
1	Re	2.548747	-0.363521	2.644203
2	Sr	0.814717	-1.321447	0.705222
3	Sr	2.252752	1.519887	0.621795
4	Re	0.509228	0.675257	2.606342

Total Energy -404.8866224 Ha

73.2 $Sr_2Re_2H_2O^-$

Final Coordinates (Angstroms)

ATOM		X	Y	Z
1	Re	2.499573	-0.313931	2.831136
2	Sr	0.697628	-1.561612	0.503413
3	Sr	2.390422	1.734119	0.409458
4	Re	0.537821	0.651600	2.833554
5	H	5.097884	1.404364	2.701033
6	H	5.757624	1.865343	1.434504
7	O	5.965184	1.765485	2.387214

Total Energy -481.9941071 Ha

Vibrational frequencies of H_2O, intensities

Mode	au_amu	cm-1	km/mol
19	0.314335	1615.8	588.82
20	0.657935	3382.1	674.22
21	0.694507	3570.1	381.19

74.1 $Sr_2Pt_2^-$

Final Coordinates (Angstroms)

ATOM		X	Y	Z
1	Pt	2.724637	0.920027	3.642273
2	Pt	0.356057	-0.267465	0.232588
3	Sr	2.773669	1.065956	0.818616

4	Sr	0.257535	-0.351857	3.067359

Total Energy -493.7049320 Ha

74.2 $Sr_2Pt_2H_2O^-$

Reaction precursor

Final Coordinates (Angstroms)

ATOM		X	Y	Z
1	Pt	2.780793	-0.141992	2.981309
2	Pt	1.258072	-0.871933	0.821281
3	Sr	2.255631	1.830345	0.911902
4	Sr	-0.182598	0.550240	3.046343
5	H	1.957029	3.275381	3.362062
6	H	3.043210	2.196947	3.419255
7	O	2.939639	3.199251	3.213680

Total Energy -570.1453842 Ha

Vibrational frequencies of H_2O, intensities

Mode	au_amu	cm-1	km/mol
19	0.306327	1574.7	92.12
20	0.521018	2678.3	406.47
21	0.631541	3246.4	673.86

Intermediate state

$coordinates

Pt	5.31833879759359	-0.29999165262617	5.67605280367074
Pt	2.37517189770864	-1.53387501282019	1.58841752790009
Sr	4.28084757008965	3.40629060589168	1.63077401556159
Sr	-0.37986633002602	1.03587106514149	5.74491467688880
H	3.20589908895601	5.77532381521068	6.39146909952306
H	5.19365249468147	2.53644161843411	6.06405159842156
O	4.96574885004477	5.48099935296760	5.70829476279480

Total Energy -570.135519 Ha

75.1 $SrCd_3^-$

Final Coordinates (Angstroms)

ATOM		X	Y	Z
1	Cd	1.867268	-0.326768	1.946244
2	Cd	2.363998	0.107923	-1.023734
3	Sr	1.712445	2.615646	0.769064
4	Cd	-0.436562	0.368500	0.117034

Total Energy -320.3707884 Ha

75.2 $SrCd_3H_2O^-$

Final Coordinates (Angstroms)

ATOM		X	Y	Z
1	Cd	1.927595	-0.431193	2.046030
2	Cd	2.444255	-0.047560	-1.176424
3	Sr	1.789353	2.986849	0.892827
4	Cd	-0.654054	0.257205	0.046176
5	H	2.973731	1.740148	3.460686
6	H	4.179245	2.536096	2.917465
7	O	3.286538	2.679407	3.310180

Total Energy -397.5100899 Ha

Vibrational frequencies of H_2O, intensities

Mode	au_amu	cm-1	km/mol
19	0.302604	1555.5	33.75
20	0.607144	3121.0	1058.36
21	0.674605	3467.8	601.84

76.1 $SrRe_3^-$

Final Coordinates (Angstroms)

ATOM		X	Y	Z
1	Re	2.064795	-0.741381	2.816061
2	Re	1.292233	-1.383486	0.668011
3	Sr	1.181113	1.744419	0.972802
4	Re	-0.244519	-0.676595	2.463754

Total Energy -505.5281316 Ha

76.2 $SrRe_3H_2O^-$

Reaction precursor

Final Coordinates (Angstroms)

ATOM		X	Y	Z
1	Re	2.213285	-0.817047	2.873282
2	Re	1.020099	-1.414065	0.581847
3	Sr	1.175810	1.746081	0.866018
4	Re	-0.037445	-0.648583	2.441943
5	H	3.167787	1.448234	2.608914
6	H	4.238705	2.084598	1.672734
7	O	3.466956	2.367524	2.200835

Total Energy -582.0460665 Ha

Vibrational frequencies of H_2O, intensities

Mode	au_amu	cm-1	km/mol
19	0.303319	1559.2	101.87
20	0.444292	2283.9	1326.59
21	0.713903	3669.8	37.01

Intermediate state
$coordinates

Re	4.17875343622035	-1.54456612703398	5.45409816427416
Re	1.94308899743723	-2.63391562893056	1.09657321622513
Sr	2.20789775174498	3.28940505273928	1.63221835213619
Re	-0.05843532822068	-1.21799204660686	4.61414694769014
H	5.37626727481137	1.31249369365322	4.82514115855421
H	7.94692537050577	3.48662968348674	3.19851959883379
O	6.39216744592282	4.12022753531623	3.94287017357702

Total Energy -582.022506 Ha

77.1 $SrPt_3^-$

Final Coordinates (Angstroms)

ATOM		X	Y	Z
1	Pt	3.074648	-0.277711	2.891057
2	Pt	1.661065	-0.779543	0.544736
3	Sr	2.585557	1.806242	1.081318
4	Pt	0.485282	0.707120	2.566796

Total Energy -637.6681041 Ha

77.2 $SrPt_3H_2O^-$
Reaction precursor

Final Coordinates (Angstroms)

ATOM		X	Y	Z
1	Pt	3.160894	-0.243201	3.020781
2	Pt	1.726575	-0.661786	0.710481
3	Sr	2.543149	1.981712	1.307108
4	Pt	0.537300	0.641105	2.821192
5	H	1.915547	2.602406	3.926863
6	H	3.287066	2.009363	3.884706
7	O	2.857645	2.929841	3.859330

Total Energy -714.1662089 Ha

Vibrational frequencies of H_2O, intensities

Mode	au_amu	cm-1	km/mol
19	0.247001	1269.7	1280.98
20	0.291111	1496.5	84.46
21	0.719961	3701.0	5.35

Intermediate state
$coordinates

Pt	5.88214777861330	-0.57613856148319	5.70455838685183
Pt	3.26482417371704	-1.14453685956047	1.25971213876015
Sr	5.12571559137898	3.71212516130523	2.67027377627200
Pt	1.11654737570192	1.15742641122821	5.41635549018998

H	1.94215005866613	3.93419491525843	6.24034747265046
H	4.44897414883158	6.24760351263411	7.93004882552663
O	3.71652654320994	6.41113542450381	6.25491406932674

Total Energy -714.167227 Ha

78.1 Tc_3Pt^-

Final Coordinates (Angstroms)

	ATOM	X	Y	Z
1	Pt	2.245366	-0.915919	1.797217
2	Tc	2.521004	0.549487	-0.292434
3	Tc	1.873252	2.642563	-0.568609
4	Tc	1.344040	1.508796	1.573635

Total Energy -524.7763099 Ha

78.2 $Tc_3PtH_2O^-$

Final Coordinates (Angstroms)

	ATOM	X	Y	Z
1	Pt	2.289755	-1.260644	1.698055
2	Tc	2.719395	0.549854	-0.150600
3	Tc	1.236581	2.365379	-0.295452
4	Tc	0.485538	0.450930	0.826210
5	H	3.051641	0.778961	3.090494
6	H	2.797006	2.177368	2.615679
7	O	3.215070	1.714314	3.369836

Total Energy -601.7654148 Ha

Vibrational frequencies of H_2O, intensities

Mode	au_amu	cm-1	km/mol
19	0.324221	1666.7	204.63
20	0.664360	3415.1	258.29
21	0.706304	3630.7	47.18

79.1 Pd_3Pt^-

Final Coordinates (Angstroms)

	ATOM	X	Y	Z
1	Pt	2.776816	-0.341203	1.169497
2	Pd	1.640156	-0.138383	-1.225952
3	Pd	2.909047	1.935585	-0.179377
4	Pd	0.598160	1.133721	0.847738

Total Energy -685.1816776 Ha

79.2 $Pd_3PtH_2O^-$

Final Coordinates (Angstroms)

ATOM		X	Y	Z
1	Pt	2.779968	-0.320245	1.189631
2	Pd	1.620208	-0.179831	-1.294720
3	Pd	2.965602	1.963545	-0.173984
4	Pd	0.558402	1.126251	0.890979
5	H	2.850847	1.626909	3.077996
6	H	2.280631	2.856098	2.484545
7	O	2.678966	2.563008	3.332041

Total Energy -762.1296131 Ha

Vibrational frequencies of H_2O, intensities

Mode	au_amu	cm-1	km/mol
19	0.320787	1649.0	363.09
20	0.681408	3502.8	167.45
21	0.700094	3598.8	44.63

80.1 Cd_4^-

Final Coordinates (Angstroms)

ATOM		X	Y	Z
1	Cd	2.367106	0.786048	1.232199
2	Cd	1.820590	-0.826233	-1.403210
3	Cd	2.124349	2.284036	-1.502117
4	Cd	-0.407383	1.000070	-0.227356

Total Energy -358.9540890 Ha

80.2 $Cd_4H_2O^-$

Final Coordinates (Angstroms)

ATOM		X	Y	Z
1	Cd	2.438606	0.810148	1.412396
2	Cd	1.853536	-1.006941	-1.528232
3	Cd	2.189552	2.421374	-1.594598
4	Cd	-0.577031	1.019340	-0.190049
5	H	3.262503	1.516524	4.011939
6	H	3.353140	2.939575	4.534187
7	O	3.426356	2.020932	4.851297

Total Energy -436.0084798 Ha

Vibrational frequencies of H_2O, intensities

Mode	au_amu	cm-1	km/mol
19	0.313998	1614.1	278.89
20	0.644675	3313.9	2616.23
21	0.720769	3705.1	59.62

81.1 Cd_3Re^-

Final Coordinates (Angstroms)

ATOM		X	Y	Z
1	Cd	2.596776	-1.037790	1.159251
2	Cd	1.852728	0.450188	-1.800567
3	Re	2.879179	1.617286	0.494621
4	Cd	0.250104	0.982036	1.021718

Total Energy -420.3878616 Ha

81.2 $Cd_3ReH_2O^-$

Final Coordinates (Angstroms)

ATOM		X	Y	Z
1	Cd	2.743322	-1.163484	1.161738
2	Cd	1.859957	0.423383	-1.863956
3	Re	2.880217	1.633796	0.530682
4	Cd	0.095291	1.118024	1.046559
5	H	3.151583	1.926670	3.783474
6	H	2.415745	2.946996	2.969724
7	O	2.800548	2.835566	3.868718

Total Energy -497.4510590 Ha

Vibrational frequencies of H_2O, intensities

Mode	au_amu	cm-1	km/mol
19	0.319294	1641.3	474.20
20	0.681477	3503.1	417.38
21	0.714956	3675.2	1.42

82.1 Cd_3Pt^-

Final Coordinates (Angstroms)

ATOM		X	Y	Z
1	Pt	2.634515	0.029781	1.152977
2	Cd	1.857244	-0.524401	-1.351682
3	Cd	3.147833	2.487489	0.149563
4	Cd	0.301739	1.245135	1.667126

Total Energy -464.5512112 Ha

82.2 $Cd_3PtH_2O^-$

Final Coordinates (Angstroms)

ATOM		X	Y	Z
1	Pt	2.092422	0.769902	0.815286
2	Cd	1.981363	-0.959448	-1.348020
3	Cd	3.862822	2.608767	-0.164540
4	Cd	-0.462532	0.633241	1.752168
5	H	2.664399	1.544451	3.111753

6	H	2.910534	2.995907	3.445557
7	O	2.897654	2.128132	3.884734

Total Energy -541.5759772 Ha

Vibrational frequencies of H_2O, intensities

Mode	au_amu	cm-1	km/mol
19	0.317811	1633.7	250.10
20	0.637453	3276.8	794.66
21	0.731231	3758.9	9.83

83.1 $CdRe_3{}^-$

Final Coordinates (Angstroms)

	ATOM	X	Y	Z
1	Cd	2.678409	-0.554421	1.564721
2	Re	1.794845	0.269179	-1.261777
3	Re	2.521300	1.974398	0.029058
4	Re	0.435540	1.348311	0.633789

Total Energy -543.4508484 Ha

83.2 $CdRe_3H_2O^-$

Final Coordinates (Angstroms)

	ATOM	X	Y	Z
1	Cd	2.729792	-0.687536	1.595168
2	Re	1.743466	0.262380	-1.166787
3	Re	2.542909	2.109563	-0.007687
4	Re	0.413928	1.353060	0.545097
5	H	3.275511	1.614756	3.697806
6	H	2.348900	2.564000	3.008568
7	O	2.892157	2.504730	3.824773

Total Energy -620.5064148 Ha

Vibrational frequencies of H_2O, intensities

Mode	au_amu	cm-1	km/mol
19	0.321627	1653.3	270.13
20	0.689244	3543.1	331.65
21	0.716374	3682.5	17.21

84.1 Sb_3Pt^-

Final Coordinates (Angstroms)

	ATOM	X	Y	Z
1	Pt	2.005906	-1.495467	-0.954948
2	Sb	1.019918	0.790156	-1.769442
3	Sb	2.589717	1.396258	0.455784
4	Sb	0.505604	-0.422737	0.880492

Total Energy -617.6913995 Ha

84.2 $Sb_3PtH_2O^-$

Final Coordinates (Angstroms)

ATOM		X	Y	Z
1	Pt	2.842212	-0.201248	1.694699
2	Sb	1.033894	0.790848	-1.601634
3	Sb	3.481796	1.356052	-0.320775
4	Sb	0.371914	-0.405728	0.851733
5	H	2.602830	1.926796	3.073015
6	H	2.722231	3.295238	2.465571
7	O	2.679747	2.873776	3.343880

Total Energy -694.7925707 Ha

Vibrational frequencies of H_2O, intensities

Mode	au_amu	cm-1	km/mol
19	0.322169	1656.1	244.91
20	0.667037	3428.9	454.46
21	0.722652	3714.8	5.35

85.1 Re_4^-

Final Coordinates (Angstroms)

ATOM		X	Y	Z
1	Re	1.794009	-0.592029	1.038661
2	Re	2.823324	0.214333	-0.855545
3	Re	1.953986	2.295293	-0.398685
4	Re	0.709238	1.417303	1.324707

Total Energy -605.0326076 Ha

85.2 $Re_4H_2O^-$

Final Coordinates (Angstroms)

ATOM		X	Y	Z
1	Re	2.008448	-0.577385	1.237947
2	Re	2.494357	0.206664	-0.911423
3	Re	2.176013	2.424683	-0.256694
4	Re	0.601738	1.280938	1.039308
5	H	3.579457	1.854326	3.253349
6	H	2.135387	2.217849	3.295192
7	O	2.951261	2.313877	3.839260

Total Energy -682.0726291 Ha

Vibrational frequencies of H_2O, intensities

Mode	au_amu	cm-1	km/mol
19	0.323041	1660.6	266.35

20	0.679296	3491.9	302.94
21	0.727551	3740.0	5.77

86.1 Re_3Pt^-

Final Coordinates (Angstroms)

	ATOM	X	Y	Z
1	Pt	2.818283	-0.670602	1.160745
2	Re	1.138422	1.431664	-0.998058
3	Re	3.332290	0.941576	-0.689479
4	Re	0.476327	-0.010391	0.619182

Total Energy -649.0686952 Ha

86.2 $Re_3PtH_2O^-$

Final Coordinates (Angstroms)

	ATOM	X	Y	Z
1	Pt	3.265861	-0.196157	1.770789
2	Re	1.278898	0.636115	-1.310947
3	Re	3.073375	1.476827	-0.167310
4	Re	0.931533	0.557614	0.969053
5	H	2.859070	1.739025	3.401660
6	H	2.015754	2.924649	3.036845
7	O	2.522171	2.582879	3.796849

Total Energy -726.0824056 Ha

Vibrational frequencies of H_2O, intensities

Mode	au_amu	cm-1	km/mol
19	0.322012	1655.3	272.57
20	0.660040	3392.9	424.59
21	0.722450	3713.7	5.76

87.1 Ir_3Pt^-

Final Coordinates (Angstroms)

	ATOM	X	Y	Z
1	Pt	3.174226	0.457974	1.683446
2	Ir	1.081716	1.021181	-0.959148
3	Ir	3.365747	1.377797	-0.594115
4	Ir	0.774030	0.141284	1.196405

Total Energy -728.7964927 Ha

87.2 $Ir_3PtH_2O^-$

Final Coordinates (Angstroms)

	ATOM	X	Y	Z
1	Pt	3.132669	-0.202508	1.177389

2	Ir	1.459637	0.149759	-1.010705
3	Ir	2.899008	2.011771	-0.111680
4	Ir	0.904406	1.039214	1.271583
5	H	2.745491	1.505546	3.471241
6	H	2.311965	2.815059	2.914478
7	O	2.493486	2.402111	3.784632

Total Energy -805.7648749 Ha

Vibrational frequencies of H_2O, intensities

Mode	au_amu	cm-1	km/mol
19	0.321040	1650.3	326.46
20	0.689406	3543.9	172.45
21	0.703761	3617.7	12.63

88.1 Pt_4^-

Final Coordinates (Angstroms)

	ATOM	X	Y	Z
1	Pt	1.650352	0.698246	1.406300
2	Pt	1.200700	-1.317543	-0.257915
3	Pt	1.795708	1.098140	-1.216264
4	Pt	-0.547270	0.646979	-0.078130

Total Energy -781.1290761 Ha

88.2 $Pt_4H_2O^-$

Final Coordinates (Angstroms)

	ATOM	X	Y	Z
1	Pt	2.708127	-0.507090	2.567906
2	Pt	0.722176	-1.062397	0.756629
3	Pt	2.231178	1.130212	0.477777
4	Pt	0.399651	0.821836	2.513021
5	H	2.885865	1.969951	3.897743
6	H	2.522758	2.903140	2.804437
7	O	2.826368	2.936330	3.735602

Total Energy -858.0822225 Ha

Vibrational frequencies of H_2O, intensities

Mode	au_amu	cm-1	km/mol
19	0.321207	1651.2	305.79
20	0.692292	3558.7	153.39
21	0.701943	3608.3	2.45

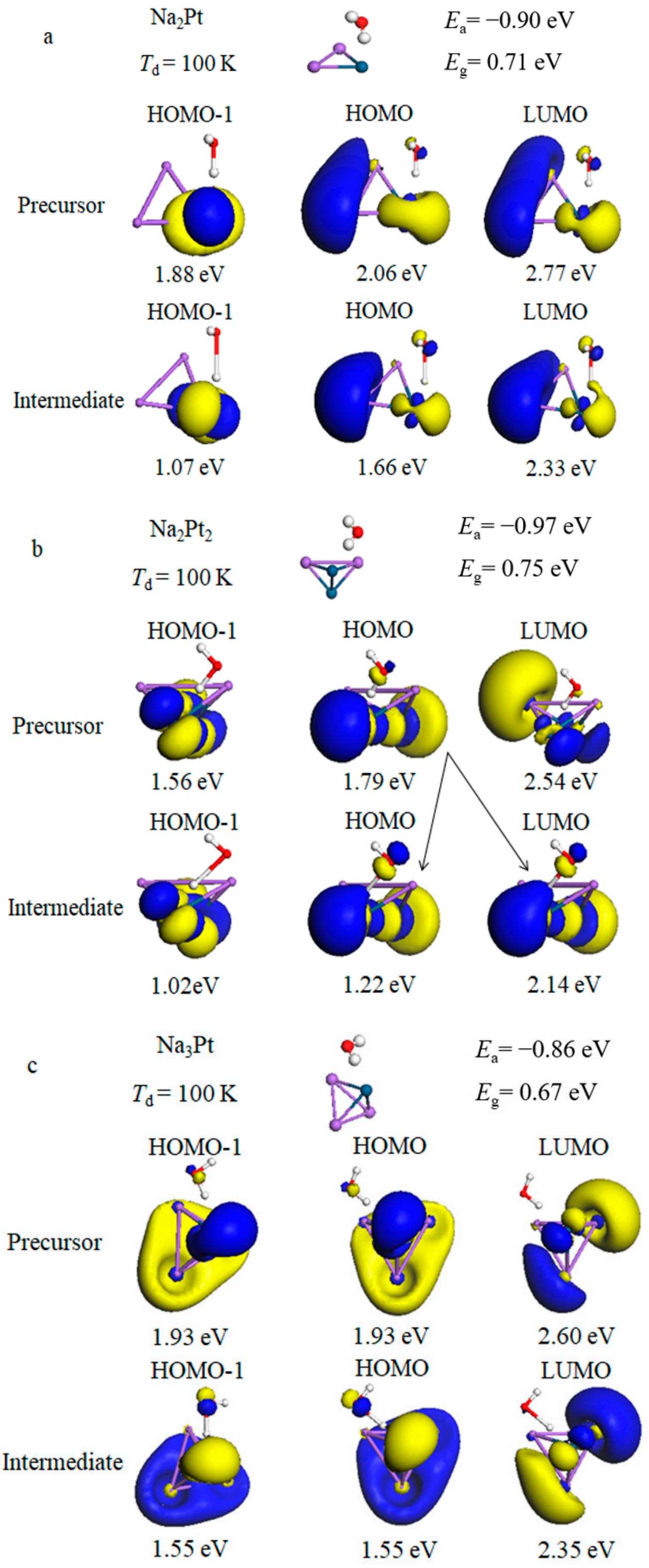
a
Na_2Pt
T_d = 100 K
E_a = −0.90 eV
E_g = 0.71 eV
HOMO-1
HOMO
LUMO
Precursor
1.88 eV
2.06 eV
2.77 eV
HOMO-1
HOMO
LUMO
Intermediate
1.07 eV
1.66 eV
2.33 eV
b
Na_2Pt_2
T_d = 100 K
E_a = −0.97 eV
E_g = 0.75 eV
HOMO-1
HOMO
LUMO
Precursor
1.56 eV
1.79 eV
2.54 eV
HOMO-1
HOMO
LUMO
Intermediate
1.02eV
1.22 eV
2.14 eV
c
Na_3Pt
T_d = 100 K
E_a = −0.86 eV
E_g = 0.67 eV
HOMO-1
HOMO
LUMO
Precursor
1.93 eV
1.93 eV
2.60 eV
HOMO-1
HOMO
LUMO
Intermediate
1.55 eV
1.55 eV
2.35 eV

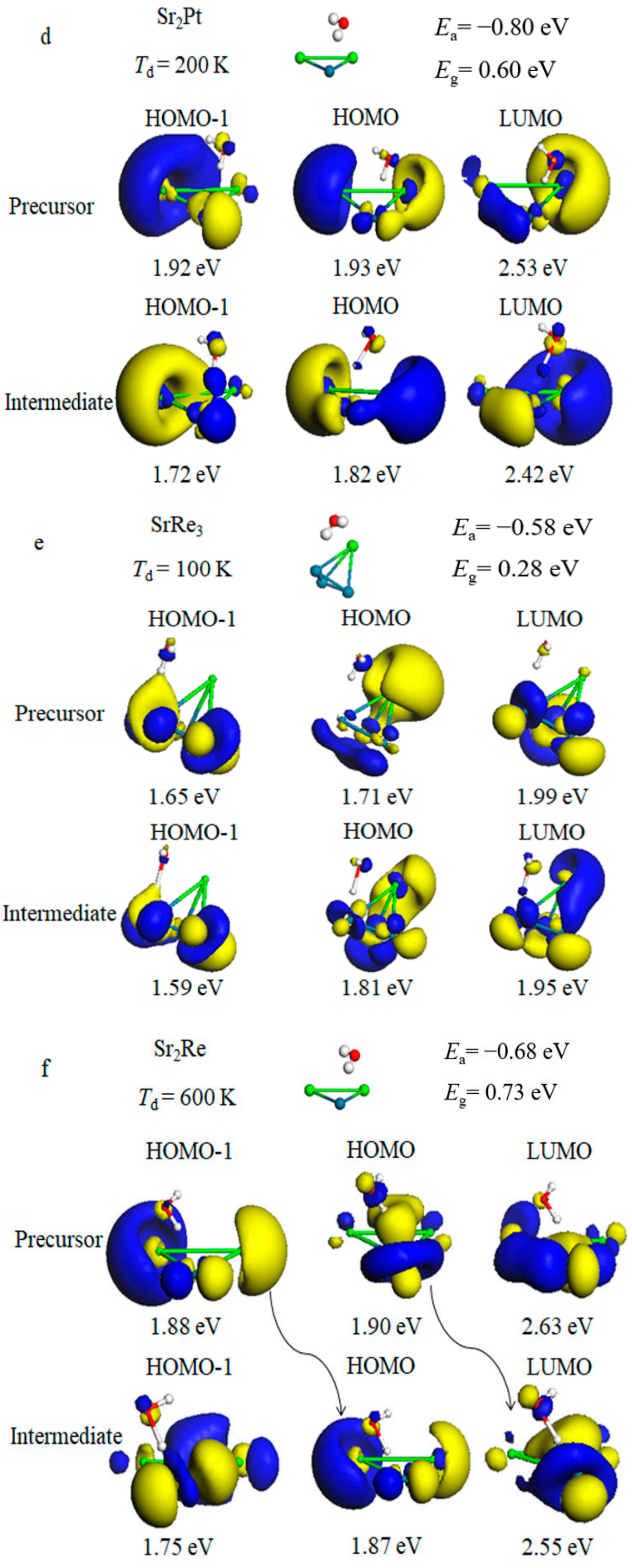
d
Sr2Pt
Td = 200 K
Ea = −0.80 eV
Eg = 0.60 eV
HOMO-1
HOMO
LUMO
Precursor
1.92 eV
1.93 eV
2.53 eV
HOMO-1
HOMO
LUMO
Intermediate
1.72 eV
1.82 eV
2.42 eV
e
SrRe3
Td = 100 K
Ea = −0.58 eV
Eg = 0.28 eV
HOMO-1
HOMO
LUMO
Precursor
1.65 eV
1.71 eV
1.99 eV
HOMO-1
HOMO
LUMO
Intermediate
1.59 eV
1.81 eV
1.95 eV
f
Sr2Re
Td = 600 K
Ea = −0.68 eV
Eg = 0.73 eV
HOMO-1
HOMO
LUMO
Precursor
1.88 eV
1.90 eV
2.63 eV
HOMO-1
HOMO
LUMO
Intermediate
1.75 eV
1.87 eV
2.55 eV

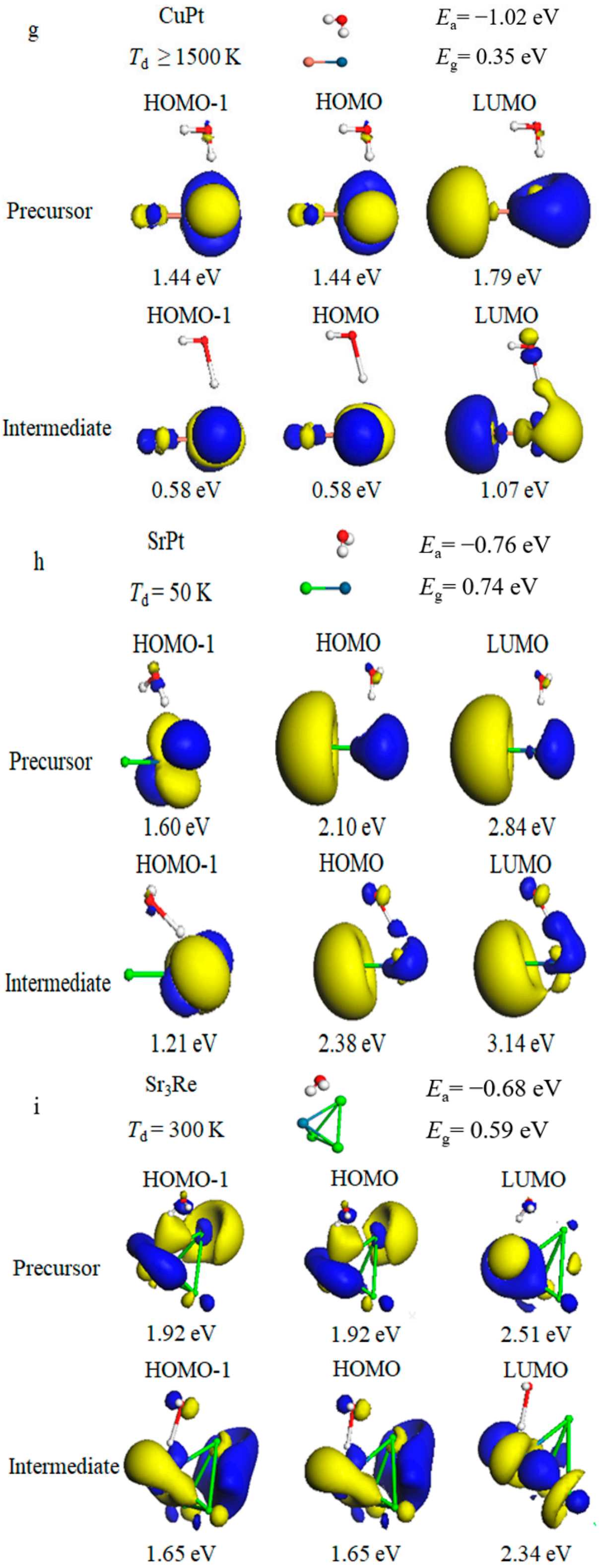
g
CuPt
$T_d \geq 1500$ K
$E_a = -1.02$ eV
$E_g = 0.35$ eV
HOMO-1
HOMO
LUMO
Precursor
1.44 eV
1.44 eV
1.79 eV
HOMO-1
HOMO
LUMO
Intermediate
0.58 eV
0.58 eV
1.07 eV
h
SrPt
$T_d = 50$ K
$E_a = -0.76$ eV
$E_g = 0.74$ eV
HOMO-1
HOMO
LUMO
Precursor
1.60 eV
2.10 eV
2.84 eV
HOMO-1
HOMO
LUMO
Intermediate
1.21 eV
2.38 eV
3.14 eV
i
Sr_3Re
$T_d = 300$ K
$E_a = -0.68$ eV
$E_g = 0.59$ eV
HOMO-1
HOMO
LUMO
Precursor
1.92 eV
1.92 eV
2.51 eV
HOMO-1
HOMO
LUMO
Intermediate
1.65 eV
1.65 eV
2.34 eV

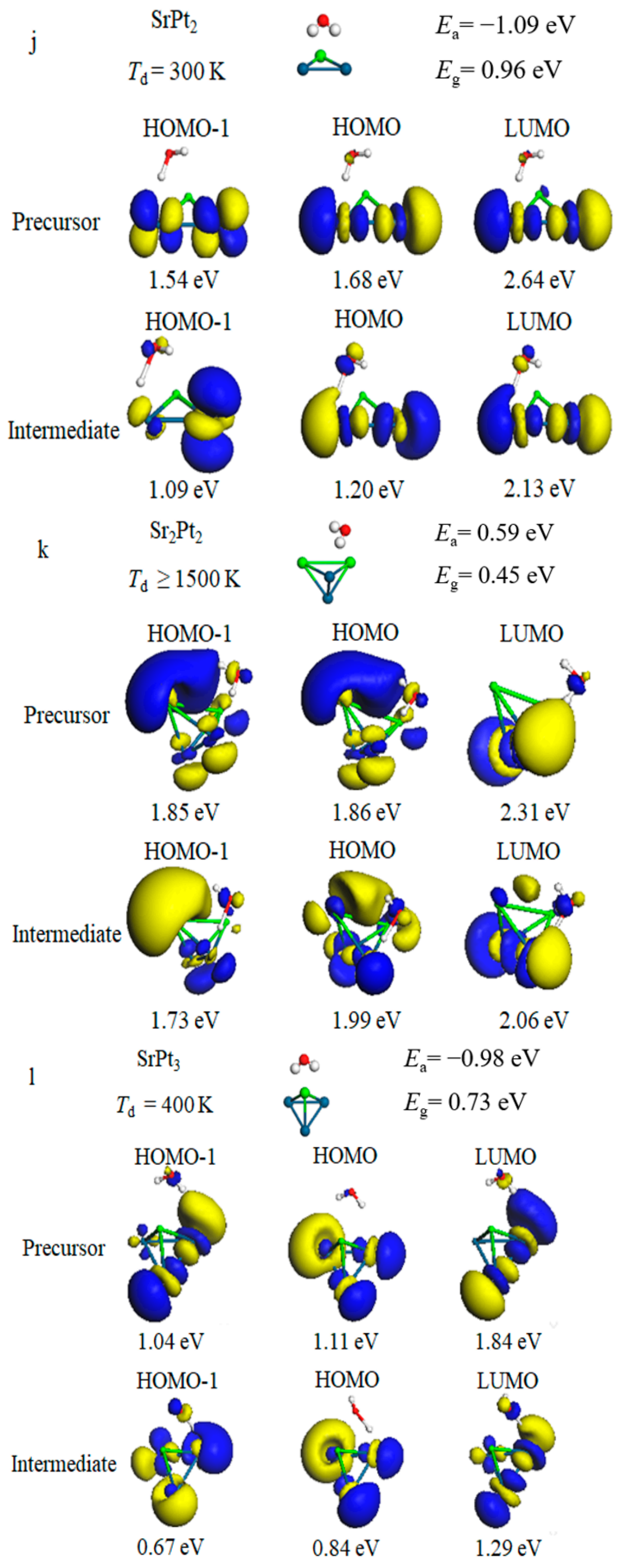
j
SrPt2
Ta= 300 K
Ea= −1.09 eV
Eg= 0.96 eV
HOMO-1
HOMO
LUMO
Precursor
1.54 eV
1.68 eV
2.64 eV
HOMO-1
HOMO
LUMO
Intermediate
1.09 eV
1.20 eV
2.13 eV
k
Sr2Pt2
Td ≥ 1500 K
Ea= 0.59 eV
Eg= 0.45 eV
HOMO-1
HOMO
LUMO
Precursor
1.85 eV
1.86 eV
2.31 eV
HOMO-1
HOMO
LUMO
Intermediate
1.73 eV
1.99 eV
2.06 eV
l
SrPt3
Td = 400 K
Ea= −0.98 eV
Eg= 0.73 eV
HOMO-1
HOMO
LUMO
Precursor
1.04 eV
1.11 eV
1.84 eV
HOMO-1
HOMO
LUMO
Intermediate
0.67 eV
0.84 eV
1.29 eV

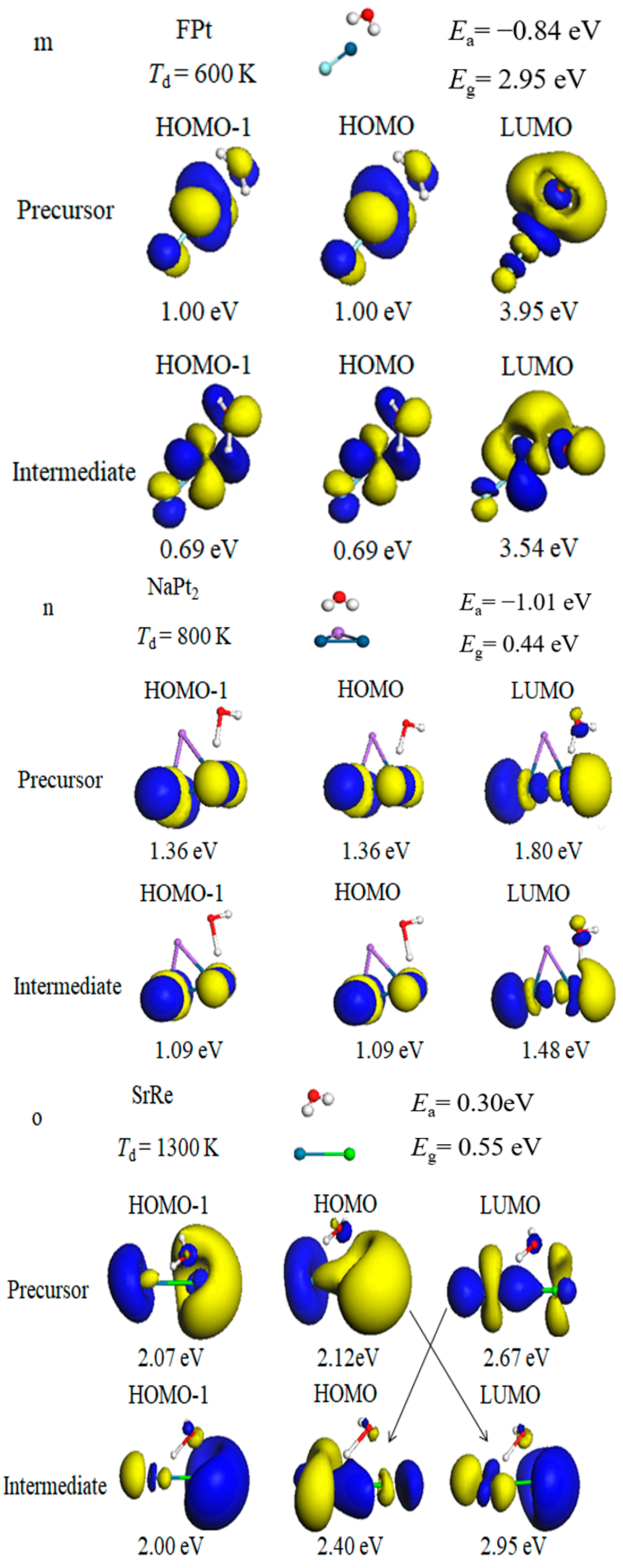

m
FPt
T_d = 600 K
E_a = −0.84 eV
E_g = 2.95 eV
HOMO-1
HOMO
LUMO
Precursor
1.00 eV
1.00 eV
3.95 eV
HOMO-1
HOMO
LUMO
Intermediate
0.69 eV
0.69 eV
3.54 eV
n
$NaPt_2$
T_d = 800 K
E_a = −1.01 eV
E_g = 0.44 eV
HOMO-1
HOMO
LUMO
Precursor
1.36 eV
1.36 eV
1.80 eV
HOMO-1
HOMO
LUMO
Intermediate
1.09 eV
1.09 eV
1.48 eV
o
SrRe
T_d = 1300 K
E_a = 0.30eV
E_g = 0.55 eV
HOMO-1
HOMO
LUMO
Precursor
2.07 eV
2.12eV
2.67 eV
HOMO-1
HOMO
LUMO
Intermediate
2.00 eV
2.40 eV
2.95 eV

Figure S1. The frontier orbitals (HOMO-1, HOMO, and LUMO) of the precursors and intermediate states of catalysts in Table 2 in the dissociation reaction along with total energy. E_a stands for adsorption energy of H_2O, E_g for LUMO-HOMO gap of the catalysts adsorbing H_2O, T_d for dissociation temperature.
